# Comparative analysis of basic helix–loop–helix gene family among *Brassica oleracea*, *Brassica rapa,* and *Brassica napus*

**DOI:** 10.1186/s12864-020-6572-6

**Published:** 2020-02-24

**Authors:** Liming Miao, Yingying Gao, Kun Zhao, Lijun Kong, Shubo Yu, Rongrong Li, Kaiwen Liu, Xiaolin Yu

**Affiliations:** 1 Laboratory of Cell and Molecular Biology, Institute of Vegetable Science, Zhejiang University, 866 Yuhangtang Road, Zhejiang, 310058 Hangzhou China; 2 Key Laboratory of Horticultural Plant Growth, Development, and Quality Improvement, Ministry of Agriculture, Zhejiang Provincial Key Laboratory of Horticultural Plant Integrative Biology, Zhejiang, 310058 Hangzhou China

**Keywords:** *Brassica* crops, bHLH gene family, Whole genome identification, Evolutionary analysis, Expression analysis

## Abstract

**Background:**

The basic helix–loop–helix (bHLH) is the second largest gene family in the plant, some members play important roles in pistil development and response to drought, waterlogging, cold stress and salt stress. The bHLH gene family has been identified in many species, except for *Brassica oleracea* and *B. napus* thus far*.* This study aims to identify the bHLH family members in *B. oleracea*, *B. rapa* and *B. napus*, and elucidate the expression, duplication, phylogeny and evolution characters of them.

**Result:**

A total of 268 bHLH genes in *B. oleracea*, 440 genes in *B. napus*, and 251 genes in *B. rapa*, including 21 new bHLH members, have been identified*.* Subsequently, the analyses of the phylogenetic trees, conserved motifs and gene structures showed that the members in the same subfamily were highly conserved. Most *Ka/Ks* values of homologous gene were < 1, which indicated that these genes suffered from strong purifying selection for retention. The retention rates of *BrabHLH* and *BolbHLH* genes were 51.6 and 55.1%, respectively. The comparative expression patterns between *B. rapa* and *B. napus* showed that they had similar expression patterns in the root and contrasting patterns in the stems, leaves, and reproductive tissues. In addition, there were 41 and 30 differential expression bHLH genes under the treatments of ABA and JA, respectively, and the number of down regulation genes was significantly more than up regulation genes.

**Conclusion:**

In the present study, we identified and performed the comparative genomics analysis of bHLH gene family among *B. oleracea*, *B. rapa* and *B. napus*, and also investigated their diversity. The expression patterns between *B. rapa* and *B. napus* shows that they have the similar expression pattern in the root and opposite patterns in the stems, leaves, and reproduction tissues. Further analysis demonstrated that some bHLH gene members may play crucial roles under the abiotic and biotic stress conditions. This is the first to report on the bHLH gene family analysis in *B. oleracea* and *B. napus,* which can offer useful information on the functional analysis of the bHLH gene in plants.

## Background

*B. oleracea* L. is an extremely important cruciferous vegetable worldwide that contains rich nutrients and has a morphologically abundant variation. *B. oleracea* L. generally, includes many common foods as cultivars, including cabbage, broccoli, cauliflower, kale, Brussels sprouts, collard greens, savoy and kohlrabi. Some varieties also have excellent ornamental properties. Many gene families have been identified in *Brassica* crops belonging to the U triangle since the completed genome sequencing of many species [1]. However, the basic helix–loop–helix (bHLH) gene family has not been identified in *B. oleracea and B. napus* up to now. In our previous study, we found that the bHLH family genes may be related to the development of pistil in turnips. The analysis of the bHLH gene family is also intended to provide some clues to the functional research of its members.

The bHLH gene family was named from its bHLH domain. This domain is composed of 50–60 amino acids that can be divided into basic amino acids with 10–15 amino acids in the N-terminal and HLH region of approximately 40 amino acids in the C-terminal [2, 3]. The bHLH transcription factors generally function as homodimers or heterodimer and interact with the E-Box (5′–CANNTG-3′), most commonly G-Box (5′-CACGTG-3′), which is the *cis*-element of the gene promoter region [4]. The number of bHLH family members is extremely large, which is only next to MYB transcription factors. In the previous study, 133 bHLH genes were classified into 12 subfamilies in *Arabidopsis*; with the determination of new bHLH genes, the family was divided into 21 subfamilies [5, 6]. A total of 162 *Arabidopsis* bHLH and 167 rice bHLH genes were clustered into 25 subfamilies (A–Y) [3].

The bHLH transcription factors are also involved in many developmental processes in the plant. *LAX* (*OsbHLH164*) is expressed in the boundary between the shoot apical meristem and the region of new meristem formation and involved in the formation of all types of axillary meristems throughout the ontogeny of a rice plant [7]. Meanwhile, many bHLH genes respond to many types of stress, such as drought, salt, and cold stresses. *OsbHLH148* and *OsbHLH006* (*RERJ1*) responded to drought stress through the jasmonic acid signaling pathway [8–10]. *OrbHLH2* (a bHLH gene cloned from *Oryzaru fipogon* Griff.) overexpression could enhance salt tolerance and osmotic stress resistance in *A. thaliana* transgenic plants [11]. *OsbHLH1*, which is independent of ABA, plays a transcriptional role in cold signal transduction [12]. In addition, some bHLH genes are also involved in plant reproductive development. Silique and septum development are restricted to the basal half, and seed set is limited to the apex in the *spt-2* mutant [13]. *SPT* (*SPATULA*) and *IND* (*INDEHISCENT*) interact to mediate gynoecium and fruit development by controlling auxin distribution through cooperative binding to regulatory sequences in downstream target genes [14]. *HEC2-RNAi hec1 hec3* gynoecia lack any stigmatic development and have longer styles than the wild-type one, while pin-shaped inflorescences were observed in *HEC* overexpression lines [15]. *ETTIN* is also a negative regulator of *HEC* gene expression. *HEC* genes are possibly involved in the auxin-mediated control of gynoecium patterning [15]. Meanwhile, some bHLH genes regulate seed development, especially the color of seed coat. A loss-of-function mutant, that is, *rc* mutant, causes rice seed coat color to change from red to white [16]. *BrTT8* regulates seed coat pigment accumulation in *Brassica* crops by modulating the expression of the late biosynthetic genes of flavonoid [17].

Many bHLH families have been identified in various plant species to date. Ninety five members of the bHLH superfamily genome were classified into 19 subfamilies in peach [18]. A total of 113 bHLH transcription factors were found in strawberry [19]. bHLH genes were also identified in potato, grape, and peanut [20–22]. In *Brassica* spp., bHLH family was identified in *B. rapa*, which contains 230 bHLH transcription factors and is classified into 24 subfamilies [23]. However, the bHLH members in *B. oleracea* and *B. napus* have not been reported. In the present study, 268, 251, and 440 genes belonging to the bHLH family were identified in *B. oleracea*, *B. rapa*, and *B. napus*, respectively. Gene and protein sequences were evaluated and compared by analyzing the protein sequences, evolution of the gene family, chromosomal localization, and structure. Moreover, the expression profiles of bHLH family genes have been analyzed in different tissues and floral developmental stages to determine their function. These results will provide some useful clues for further studies on bHLH family in *Brassica* crops.

## Results

### Identification and phylogenetic tree analysis of bHLH genes

According to the BLAST results and domain verification, we obtained 268 bHLH genes in *B. oleracea* (Table S1), 440 genes in *B. napus* (Table S2, Table S3), and 251 bHLH members in *B. rapa*, while a previous study reported 230 identified bHLH genes in *B. rapa* (Table S4) [23]. The newly identified genes in *B. rapa* and their names are listed in Table S4. All bHLH genes were named based on their gene ID (Table S1, S2, S3 and S4). We constructed an unrooted cladogram with domain sequences of all bHLH genes of *B. oleracea*, *B. rapa*, *B. napus* and *A. thaliana* (Figure S1), and branches of the bHLH genes in *A. thaliana* and *B. rapa* were labeled in red and green, respectively*.* We also constructed NJ phylogenetic trees by using the domain sequences of bHLH genes from *B. oleracea*, *B. rapa*, and *B. napus*, respectively (Figure S2, Figure S3, Figure S4, Figure S5). These results will help us to classify different subfamilies of bHLH genes of the three *Brassica* crops.

### Analysis of conserved short amino acid sequence and structure

We searched 15 conserved motifs of all bHLH members from *B. oleracea*, *B. rapa* and *B. napus* by using MEME, and the motifs were visualized with sequence logo plot (Figure S6). All conserved motifs were with NJ phylogenetic trees, as shown in Figures S7, Figure S8, Figure S9 and the different colors represent different motifs. Then, we divided all bHLH genes into different subfamilies according the phylogenetic trees and conserved motifs. We used three methods to determine the subfamily of bHLH genes in *B. oleracea* and *B napus*. The first one, according to the subfamiles of bHLH genes in *A. thaliana* and *B. rapa*, the subfamilies of newly identified bHLH genes in *B. oleracea, B. rapa* and *B. napus* were classified (Figure S1). The second one, we classified the newly identified bHLH genes by the distance between the branches of their phylogenetic trees (Figures S2, Figure S3, Figure S4. Figure S5). The third one, we classified them according to the conservative motifs (Figures S7, Figure S8, Figure S9). Finally, the bHLH genes from *B. oleracea* and *B. rapa*, *B. napus* AA genome, and *B. napus* CC genome were classified into 25, 26, 23, and 22 subfamilies, respectively. *B. oleracea* had the same subfamilies, and *B. rapa* was divided into Ib (1) and Ib (2) in *B. rapa* except subfamily Ib. Subfamilies X and XIV had no members in *B. napus* AA genome, while subfamilies X, XIV, and IIIb had no members in *B. napus* CC genome. In *B. oleracea*, the largest subfamily was Ib, which had 32 members, and the smallest was orphan, which had one member. Subfamily XII was the largest one in *B. rapa* and *B. napus*, while subfamilies IVd and XIV were the smallest subfamilies in *B. rapa.* Subfamilies IVd and IIIf were the smallest subfamilies in the *B. napus*, which had one and six members, respectively.

For the conserved short amino acid sequence analysis, two motifs (motif 1 and motif 2) were highly conserved throughout all bHLH genes. Genes on adjacent evolutionary branches had highly conserved motifs in *B. oleracea*, *B. rapa*, and *B. napus*. Highly conserved motifs were found in the same subfamily among the three *Brassica* crops. For example, subfamily III (d + e) had the same conserved motifs (motif 1, 2, 4, 5, 8, and 12) in three *Brassica* crops. Results indicated that motifs 1 and 2 were the most conserved motifs in all bHLH genes, and the motifs in the same subfamily from different *Brassica* crops were highly conserved.

To determine whether the number of exons and introns in different subfamilies is conserved, we analyze the intron–exon location of all bHLH members from three *Brassica* crops and integrated the results according to the NJ phylogenetic trees, as shown in Figure S10, Figure S11 and Figure S12. In *B. oleracea*, most members in some subfamilies had the similar gene structure. For instance, most of genes in subfamilies VIIIb and III(d + e) had one exon and no intron. However, the numbers of some other subfamilies were diverse. For instance, subfamily Ib was the largest subfamily. In this subfamily, 16 members had 3 exons and 2 introns, 13 of which had 2 exons and 1 intron; one gene had 1 exon and without intron, *BolbHLH089* had four exons and three introns, and *BolbHLH255* have six exons and five introns. *B. rapa* and *B. napus* showed similar characters with some subfamilies conserved, while some were diverse. For the conserved subfamily (III(d + e)) in different *Brassica* crops, the gene structures of most members were conserved. In contrast to the conservation in short amino acid sequence, the gene structures in the same subfamilies were not highly conserved.

### Molecular characteristics analysis and chromosomal localization of bHLH genes in *B. oleracea, B. rapa*, and *B. napus*

We analyzed the molecular characteristics of bHLH genes in *B. oleracea*, *B. rapa*, and *B. napus* (Table S1, S2, S3 and S4). In *B. oleracea*, the ORF length of all bHLH genes ranged from 273 bp (*BolbHLH143*) to 2985 bp (*BolbHLH063* and *BolbHLH114*), and the encoding 91~994 amino acids, respectively. The gene with the largest molecular weight was BolbHLH063 (112.881 kDa), while the minimum molecular weight of all genes was 10.253 kDa (BolbHLH143). The theoretical isoelectric point (pI) ranged from 4.49 (BolbHLH072) to 10.26 (BolbHLH250). The pI of 174 deduced proteins were < 7 (64.9%), thereby indicating that these encoded proteins were acidic proteins and the rest 35.1% were basic proteins (Table S1). In *B. rapa*, the ORF length of all bHLH genes ranged from 255 bp (*BrabHLH067*) to 3606 bp (*BrabHLH198*), and the minimum to maximum molecular weight was 9.799 kDa (BrabHLH067) to 132.383 kDa (BrabHLH198). Among all *BrabHLH* deduced proteins, the pI of 156 proteins (62.2%) were < 7, and the pI of the other 95 proteins (37.8%) were > 7. In *B. napus*, the ORF length of all bHLH genes ranged from 243 bp (*BnabHLH020*) to 4320 bp (*BnabHLH017*). The pI of 295 deduced proteins (67.0%) were < 7, including 141 proteins in the AA genome (65.3%) and 154 proteins in CC genome (68.8%), and the pI of other 145 proteins were > 7.

**Fig. 1:**
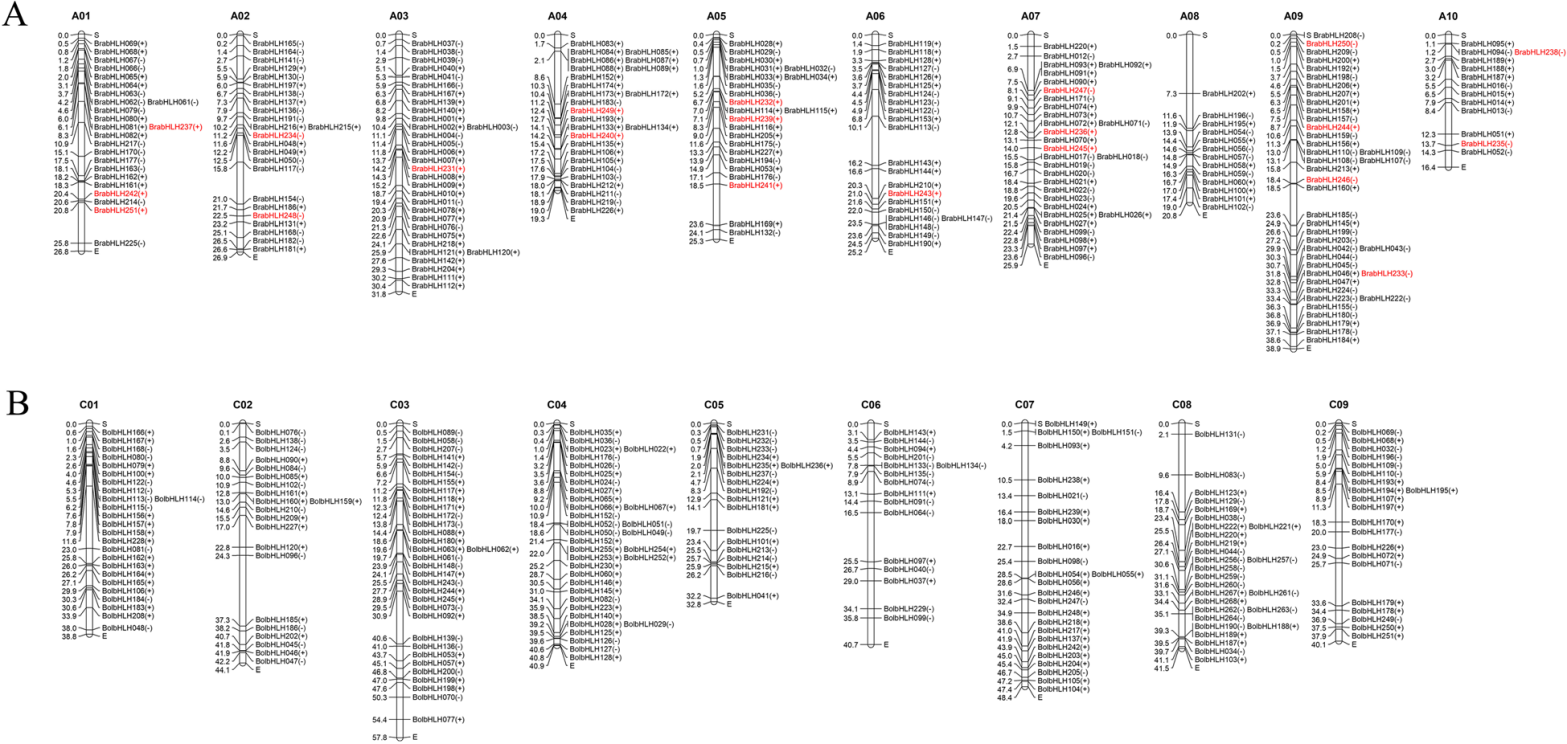
Chromosome location of bHLH genes in *B. rapa* (**a**) and *B. oleracea* (**b**). The symbols + and − indicate the gene located in sense or antisense strands, respectively. The genes in red are the newly identified bHLH genes in *B. rapa*

**Fig. 2:**
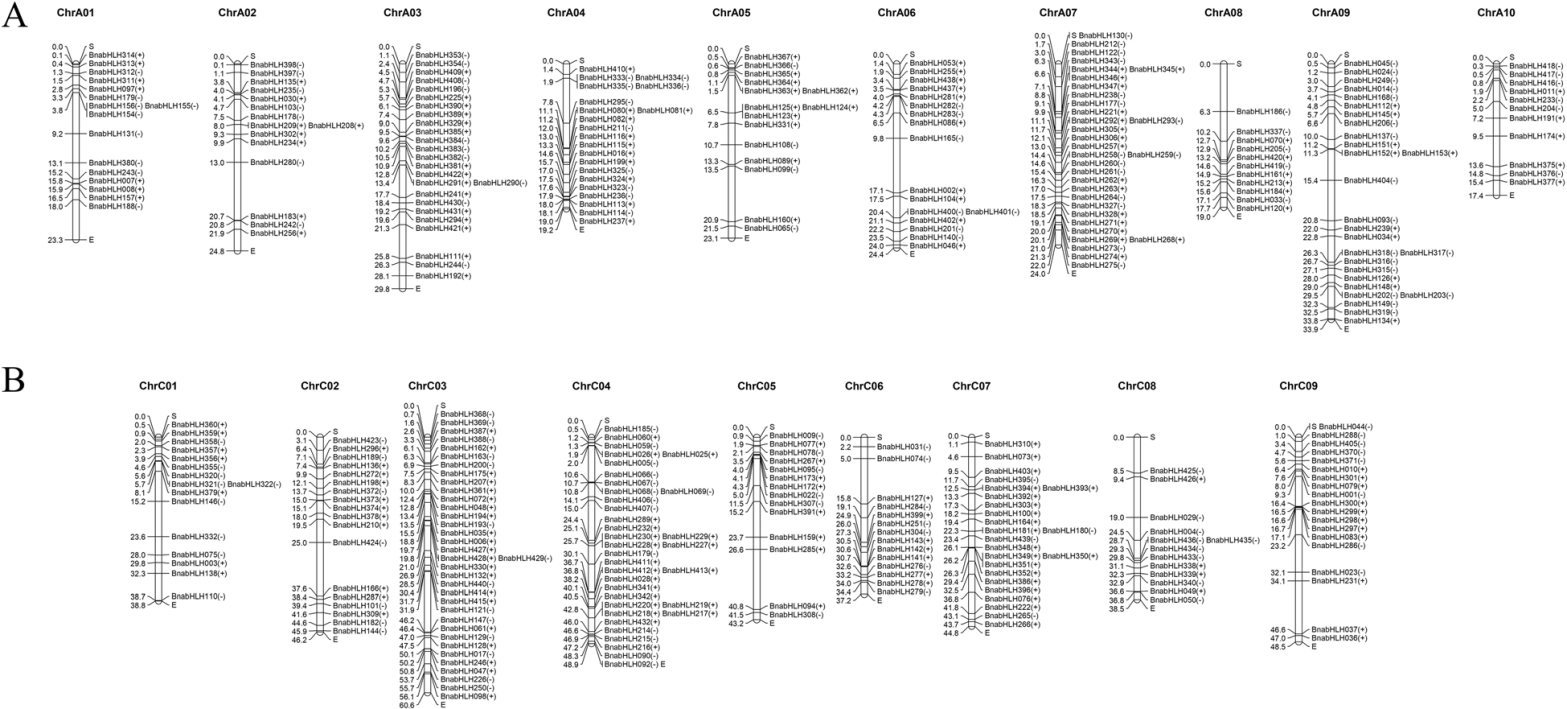
Chromosome location ofAA genome of *B. napus* (**a**), and CC genomes of *B. napus* (**b**). The symbols + and − indicate the gene located in sense or antisense strands, respectively

In *B. rapa*, 247 bHLH genes were distributed on 10 chromosomes (i.e., A01–A10) (Fig. 1a). For the 21 newly identified genes, *BrabHLH237*, *BrabHLH242*, and *BrabHLH251* were distributed on A01 chromosomes; *BrabHLH234* and *BrabHLH248* were distributed on A02; and *BrabHLH*231 and *BrabHLH*243 were distributed on A03 and A06. Two genes were located on A04 and A10, three genes were distributed on A05 and A07, and four members were mapped on A09. A total of 222 *BolbHLH* genes were distributed in 9 chromosomes (i.e., C01–C09) (Fig. 1b). The location of all *BolbHLH* genes on the chromosome can be found in Table S1. The number of bHLH genes distributed on C06 was the least, with only 16 genes, while 35 genes were distributed on C04. A total of 33 genes were distributed on the C03 chromosome, 28 genes on C08, 25 genes on C01 and C07, 21 genes on C02 and C09, and 18 genes on C05. Finally, 46 *BolbHLH* genes were distributed on different scaffolds. We also mapped the bHLH genes from the AA and CC genomes of *B. napus* (Fig. 2). For *B. napus* AA genome, 190 bHLH genes were mapped on 10 chromosomes (A01–A10). A07 had the most members (32 genes), while A08 and A10 had the minimum number of bHLH genes (11 genes). For the CC genome, 188 bHLH genes were distributed on 9 chromosomes (i.e., C01–C09). C03 and C04 had the maximum number of bHLH genes (i.e., 35 genes), and the number of bHLH genes distributed on C08 was the least, with only 13 members.

### *Ka* and *K*_*S*_ calculation of orthologous bHLH genes between *A. thaliana* and *Brassica* crops

**Fig. 3:**
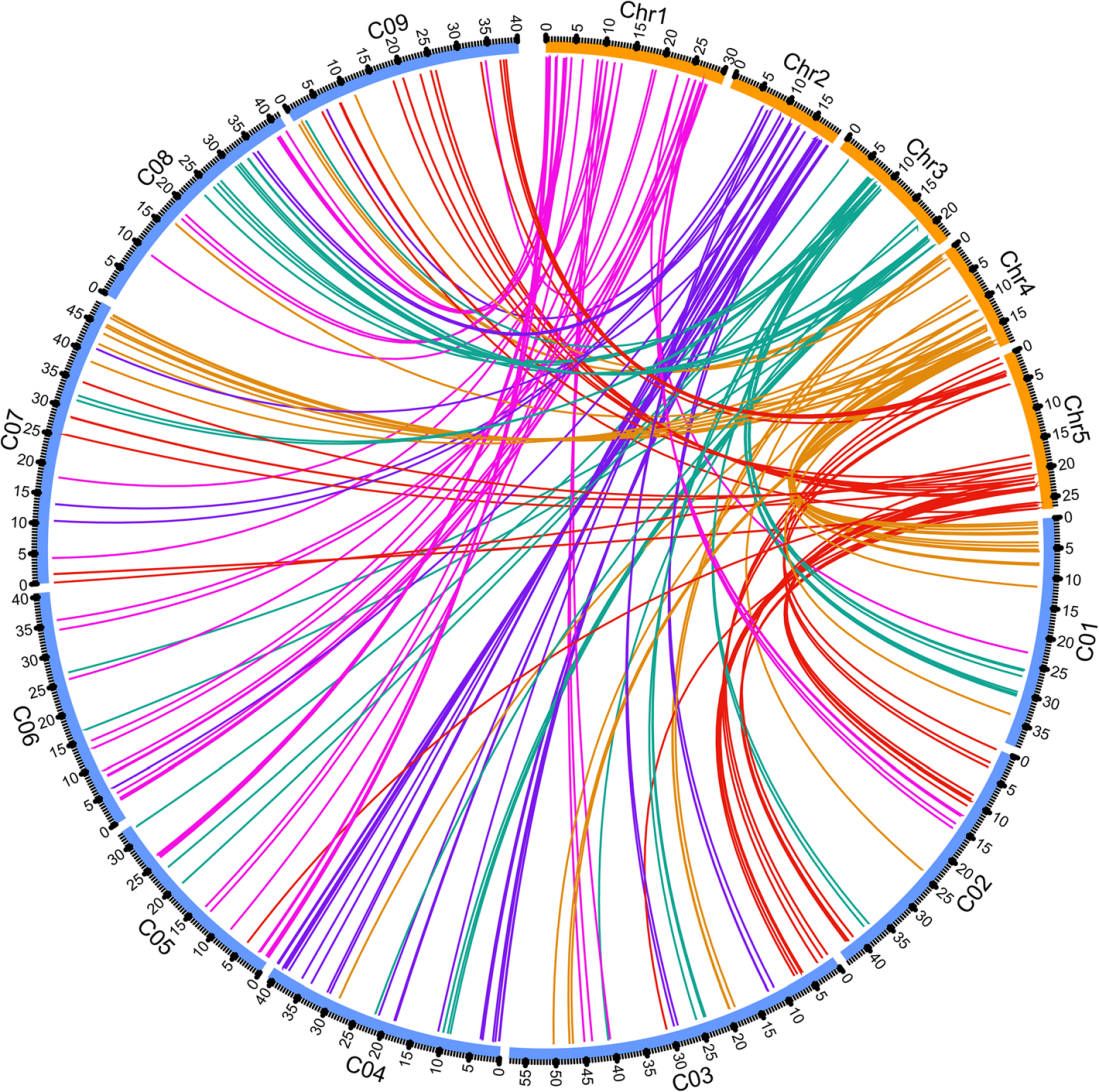
Nine *B. oleracea* (C01–C9) and five *Arabidopsis* chromosome (Chr1–Chr5) maps were based on orthologous pair positions and demonstrated highly conserved synteny. The curves of rose red, purple, green, orange, and reddish red link the bHLH gene on the Chr1, Chr2, Chr3, Chr4, and Chr5 chromosomes of *A. thaliana* and its orthologous genes in *B. olerace*, respectively

Firstly, we searched orthologous bHLH genes between *A. thaliana* and three *Brassica* crops. The *Ka*, *Ks*, and their ratio *Ka/Ks* values of 205 pairs, 206 pairs and 219 pairs orthologous bHLH genes between *A. thaliana* and *B. oleracea*, *B. rapa*, *B. napus* were obtained, respectively (Table S5, S6, S7). The results showed that most of *Ka/Ks* values of the orthologous bHLH genes between *A. thaliana* and *Brassica* crops were < 1, thereby indicating that the orthologous genes suffered from strong purifying selection for retention. A total of 13, 1, and 16 pairs of genes had the *Ka/Ks* value of > 1 in *B. oleracea*, *B. rapa* and *B. napus*, which suffered from positive Darwinian selection. For *B. oleracea,* we linearized 211 pairs of ortholog genes distributed on the chromosomes by using the Circos program (Fig. 3). For the bHLH gene family, chromosome Chr1 had additional orthologous genes with chromosomes C05 and C06 in *B. oleracea*. Chr2 and C04, Chr4 and C01, Chr5 and C02, and C03 had additional orthologous genes. The orthologous bHLH genes on Chr3 were dispersed on *B. oleracea* chromosomes.

**Fig. 4:**
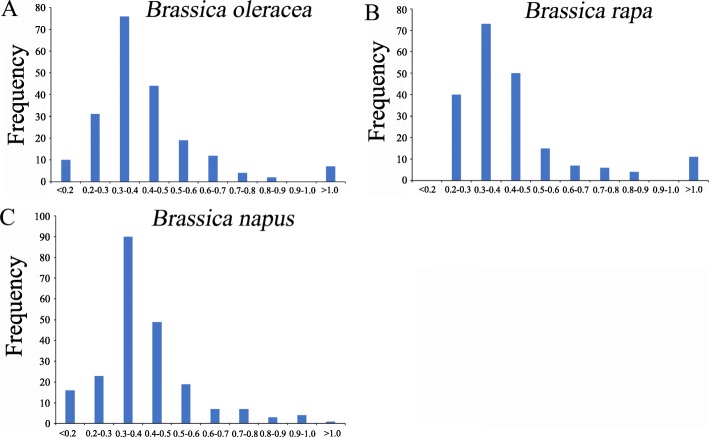
*Ks* value distribution of orthologous bHLH genes between *A. thaliana* and *B. oleracea* (**a**), *A. thaliana* and *B. rapa* (**b**), and *A. thaliana* and *B. napus* (**c**). The vertical axis indicates the frequency of paired sequences. The peaks of *Ks* value appeared between 0.3 and 0.4

The *Ks* values of the orthologous genes can be used to calculate the divergence time between *B. oleracea* and *A. thaliana*. We first counted the distribution frequency of the *Ks* value and then calculated the divergence time on the basis of the neutral substitution rate of 1.5 × 10^− 8^ substitutions per site per year for *Chs* [24]. In the present study, the *Ks* values had a concentrated location between 0.3 and 0.4 in *B. oleracea*, *B. rapa* and *B. napus* (Fig. 4). This result indicated that the divergence time of the bHLH gene family between the three *Brassica* crops and *A. thaliana* was approximately 1.0–1.3 MYA.

### Retention rates analysis of bHLH genes of three *Brassica* crops

**Fig. 5:**
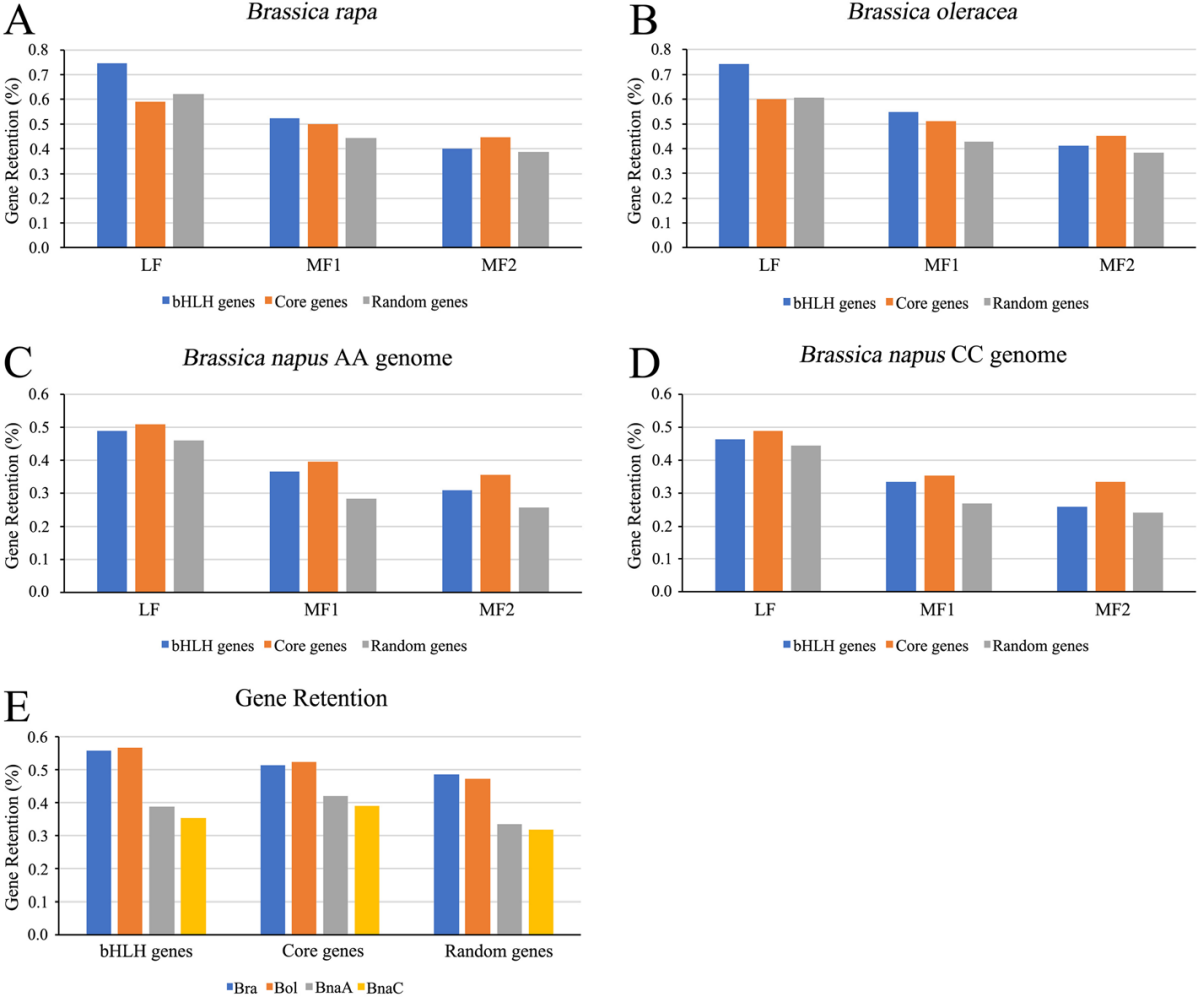
Retention rates of Arabidopsis bHLH, core, and random genes in *B. oleracea*, *B. rapa* and *B. napus.* The retention rates in the different subgenomes in (**a**) *B. rapa*, (**b**) *B. oleracea*, (**c**) *B. napus*, and (**d**) *B. napus.* (**e**) The retention rates of Arabidopsis bHLH, core, and random genes in the whole genome of *B. oleracea*, *B. rapa*, and *B. napus*

The retention rates of the identified *BrabHLH* and *BolbHLH* genes were 51.6 and 55.1%, respectively (251/486 and 268/486). We calculated the retention rate of the *AtbHLH* orthologous bHLH, core, and random genes on different subgenomes of varying species (Fig. 5). All genes had the highest retention rate in the LF subgenome. In the LF and MF1 subgenomes of *B. rapa*, *AtbHLH* orthologous bHLH genes had much higher retention rate than the core and random genes, but had lower retention rate than the core genes in *B. napus* AA genome (Fig. 5a, c). The same trend was observed between *B. oleracea* and *B. napus* CC genome (Fig. 5b and d)*.* In the MF2 subgenome, core genes had the highest retention rate in the three species. We also calculated the retention rates of Arabidopsis bHLH genes and determined the core and random genes in *B. rapa*, *B. oleracea* and *B. napus* (AA and CC genome). The retention rates of the *AtbHLH* orthologous genes in *B. rapa* and *B. oleracea* were higher than those in *B. napus* (AA and CC genome) at 56 and 57%, and higher than those of the core and random genes (Fig. 5e), respectively.

*B. napus* is a tetraploid crop derived from the cross between *Brassica rapa* and *Brassica oleracea.* We studied the bHLH gene loss, gain, and retention in *B. napus* after tetraploid procedure, aiming to understand whether the species had a preference for gene retention in a subgenome during the doubling process (Table 1). In theory, the number of bHLH genes in *B. napus* is the sum of bHLH genes in *B. rapa* and *B. oleracea.* In fact, the number was lower than the theoretical value due to the loss of genes. A total of 182 genes were lost, of which more genes were lost on the CC genome than in the AA genome. Some new genes were obtained in the sites without genes in *B. rapa* and *B. oleracea* genome. However, the gain of the genes was extremely rare. Only 13 genes, including 7 genes, were obtained in the MF2 sub genome. Some genes did not belong to the bHLH gene family, although they were the paralogous genes of a bHLH gene in *B. rapa* and *B. oleracea*. A total of 40 domain loss genes in *B. napus* were found in this study. Some identified genes did not have information about their distribution in the subgenomes, which we count as no hits. In this case, two no hits genes were found in *B. rapa*, 16 in *B. oleracea,* and 148 in *B. napus*. Hence, the most frequent occurrence of species in polyploidy is gene loss.

**Table 1:** Statistics of bHLH gene loss, gain, and retention in *B. napus* after tetraploid

Gene behavior	LF		MF1		MF2		Sum
	BnaA	BnaC	BnaA	BnaC	BnaA	BnaC	
Loss	39	42	30	34	16	21	182
Gain	2	3	1	0	3	4	13
Retention	61	54	48	44	39	33	279
Domain loss	9	12	3	7	4	5	40
No hits	/	/	/	/	/	/	148

### Signal peptide and subcellular localization prediction of bHLH family of proteins

In this study, we predicted the signal peptides of 268, 251 and 440 bHLH proteins in *B. oleracea*, *B. rapa*, and *B. napu*, respectively. Only one and two members had signal peptides in *B. oleracea* and *B. rapa*, which were BolbHLH128 (Bol021805), BrabHLH084 (Bra014653), and BrabHLH168 (Bra029354, Figure S13). According to the C, S, and Y values, the site near serine may be a potential signal peptide shear site.

**Fig. 6:**
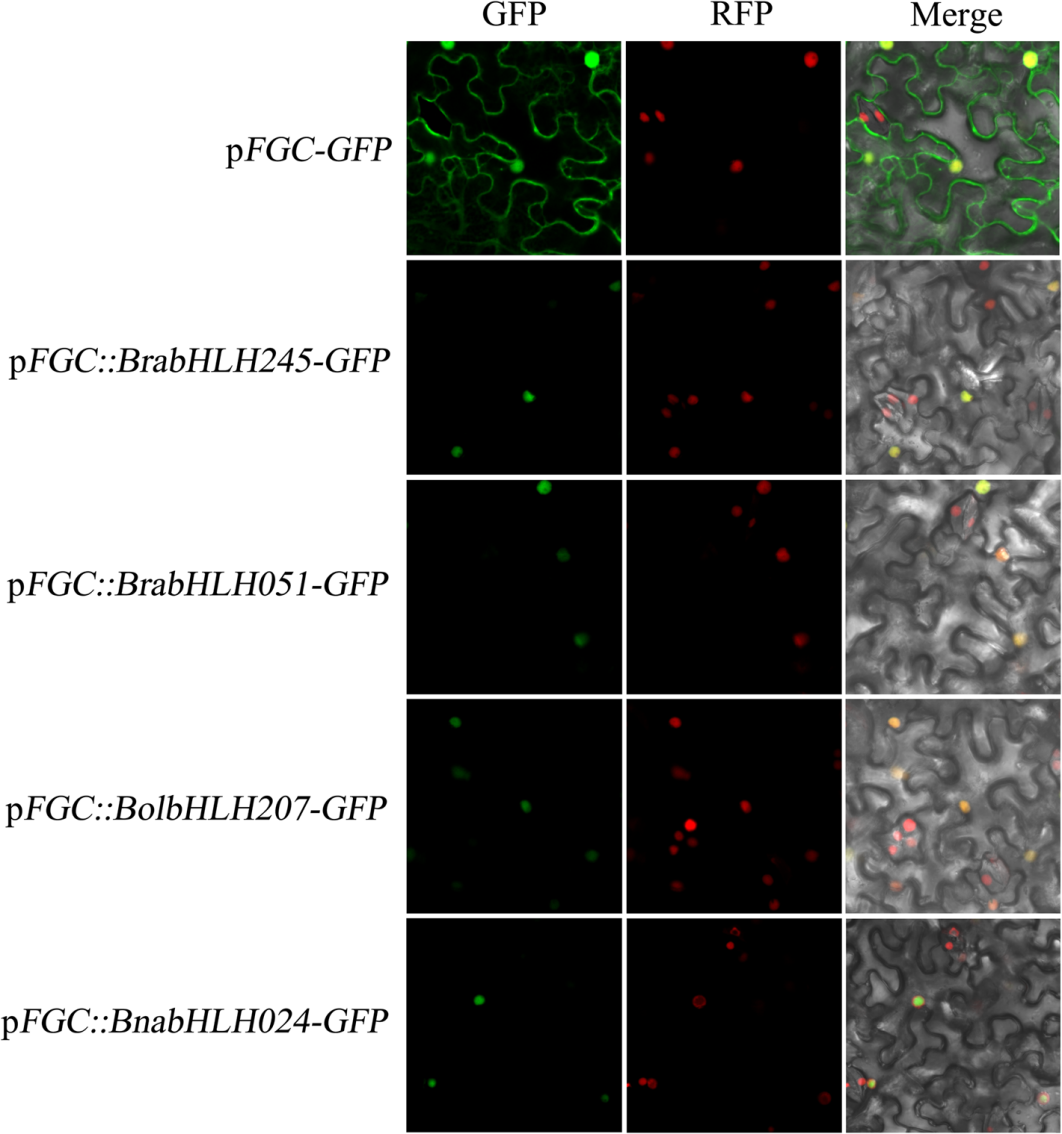
Subcellular localization of four selected bHLH genes in tobacco cells

To determine the subcellular localization of all bHLH proteins in the cell, we performed prediction analysis by three methods. Genes whose prediction results were inconsistent or not located in the nucleus were shown in Table S8. If the two or more of the predicted results are identical, it has been regarded as the subcellular localization of the encoded protein. With the exceptions of the genes in Table S8, all the other genes were predicted to be located in the nucleus by three methods. These results show that most of the bHLH genes were located in the nucleus, while some were located in other organelles. For example, BrabHLH037 was predicted to be located in chloroplast, cytoplasm, BrabHLH155 located in chloroplast and BolbHLH188 located in golgi apparatus. There are three different prediction results of 6 genes, which are marked as not sure in Table S8. We selected 4 genes (BrabHLH245, BrabHLH051, BolbHLH207 and BnabHLH024) randomly from the Table S8 for instantaneous expression in tobacco cells, and the results showed that all of these proteins were located in the nucleus (Fig. 6). This result indicates that the predictions of subcellular localization of bHLH proteins obtained by the three methods are reliable.

### Gene function annotation of bHLH proteins in *B. oleracea*

**Fig. 7:**
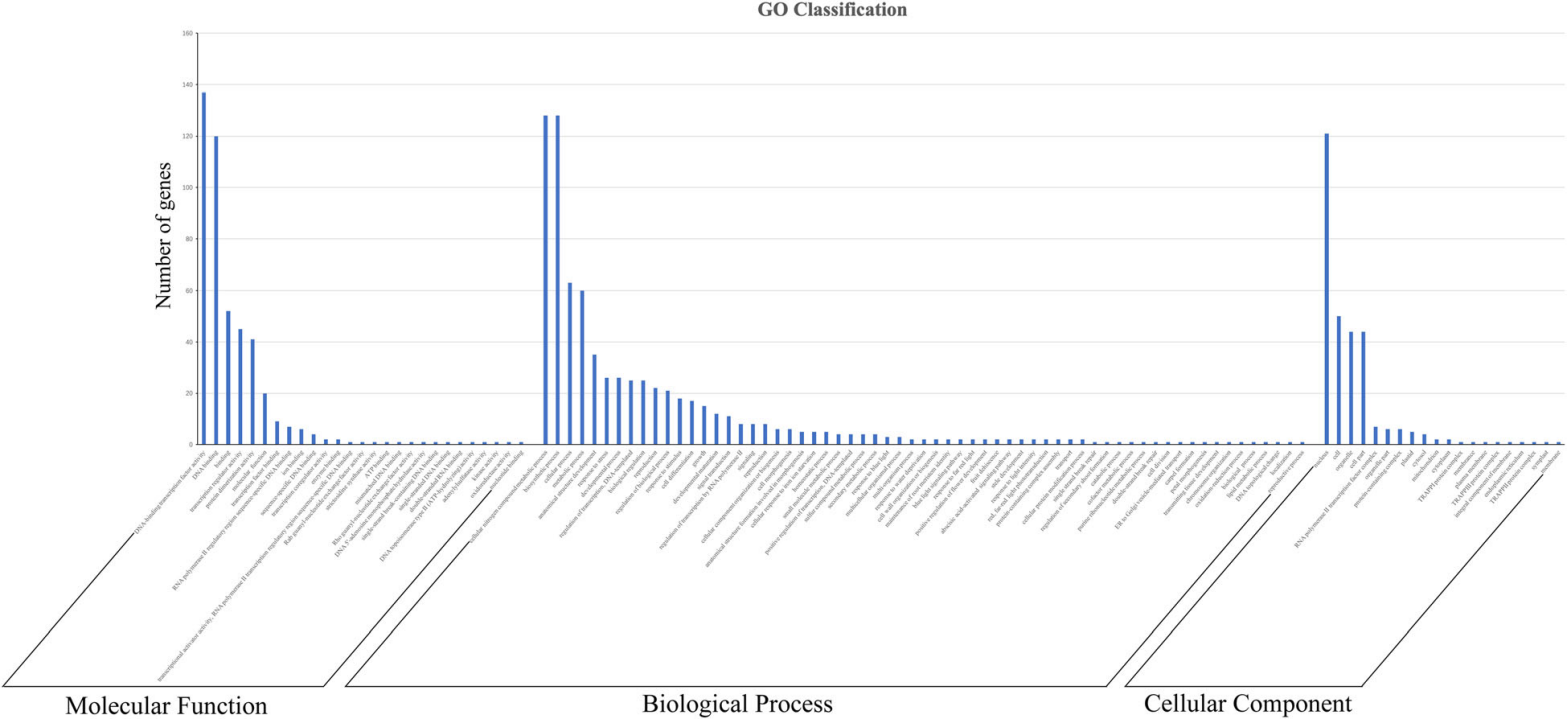
GO annotation of bHLH genes in *B. oleracea* with Blast2GO

We performed BLAST and GO annotation of 268 bHLH proteins with Blast2go (Fig. 7). GO annotation (BLAST) was utilized in this study, and only one member cannot blast to the eclectic database (BolbHLH139). Finally, 267 members obtained their own function annotation. In the molecular function, most of the gene annotations were focused on DNA-binding transcription factor, DNA-binding, and protein dimerization activities. In the biological process, most annotations were concentrated in the cellular N compound metabolic, biosynthetic, and cellular processes. In the cellular component, most gene annotations centered on nucleus, and this result was consistent with the subcellular localization prediction (Fig. 7 and Table 1).

### Gene expression analysis of bHLH gene family in *B. oleracea, B. rapaand B. napus*

**Fig. 8:**
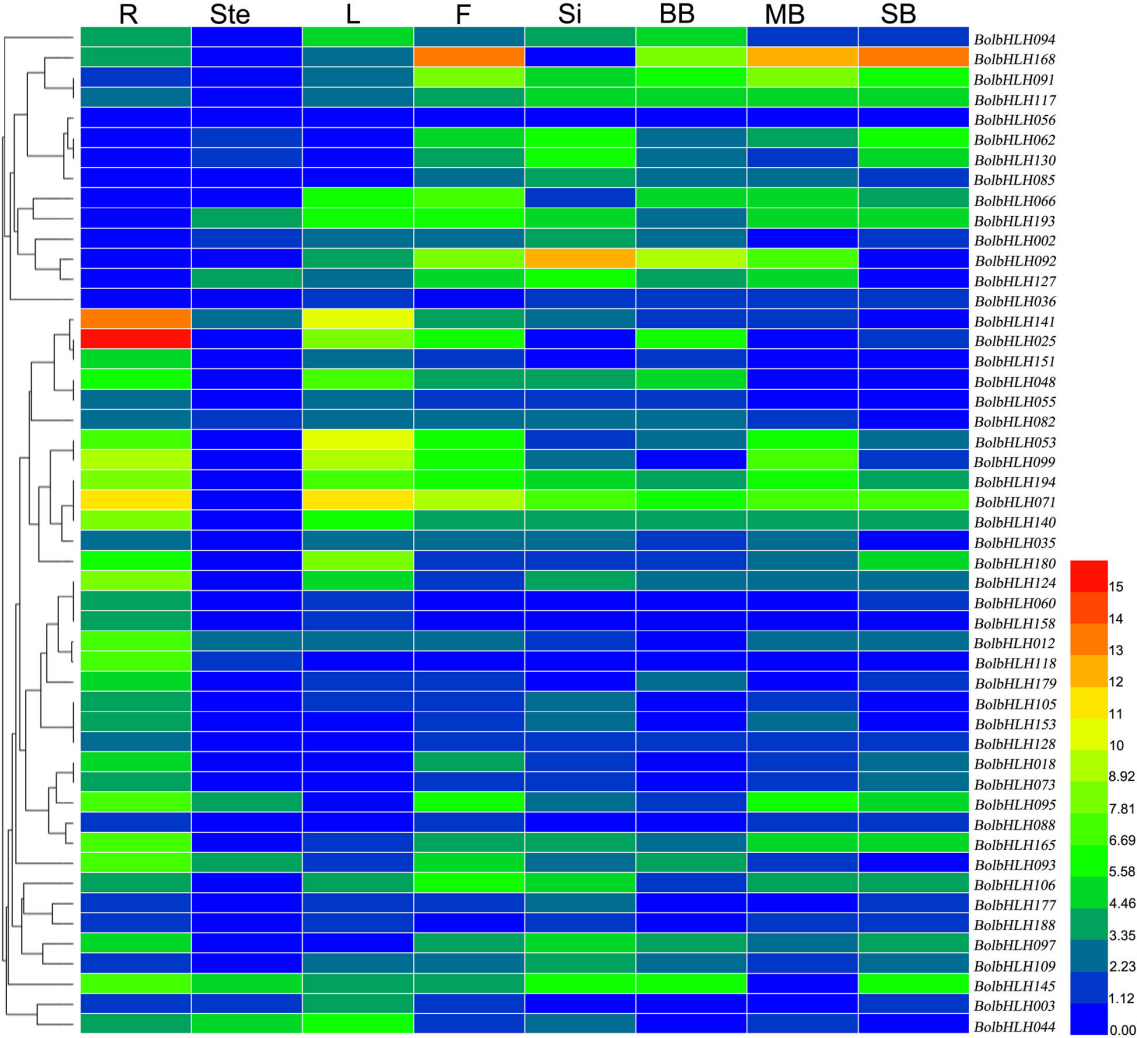
Expression pattern of 50 bHLH genes from different subfamilies in varying tissues in *B. oleracea.* All values underwent logarithmic transformation. R: root, Ste.: stem, L: leaves, F: flower, Si: silique, LB: large bud, MB: middle buds, and SB: small bud

We analyzed the temporal and spatial expression patterns of 50 *BrabHLH*, 50 *BolbHLH* and 65 *BnabHLH* genes in different tissues or organs. The original Ct values were showed in Table S9. All the relative expression results underwent logarithmic transformation. In *B. oleracea*, the expression patterns of the bHLH gene family were diverse. Some genes were highly expressed in vegetative tissues, while others were highly expressed in reproductive tissues (Fig. 8). For example, *BolbHLH053* had extremely high expression in the root and almost no expression in the pods and buds. By contrast, *BolbHLH239* had a low expression in the root, stem, and leaf and high expression in the flower, middle buds, and small buds. The expression level decreased again with reproductive organ development.

**Fig. 9:**
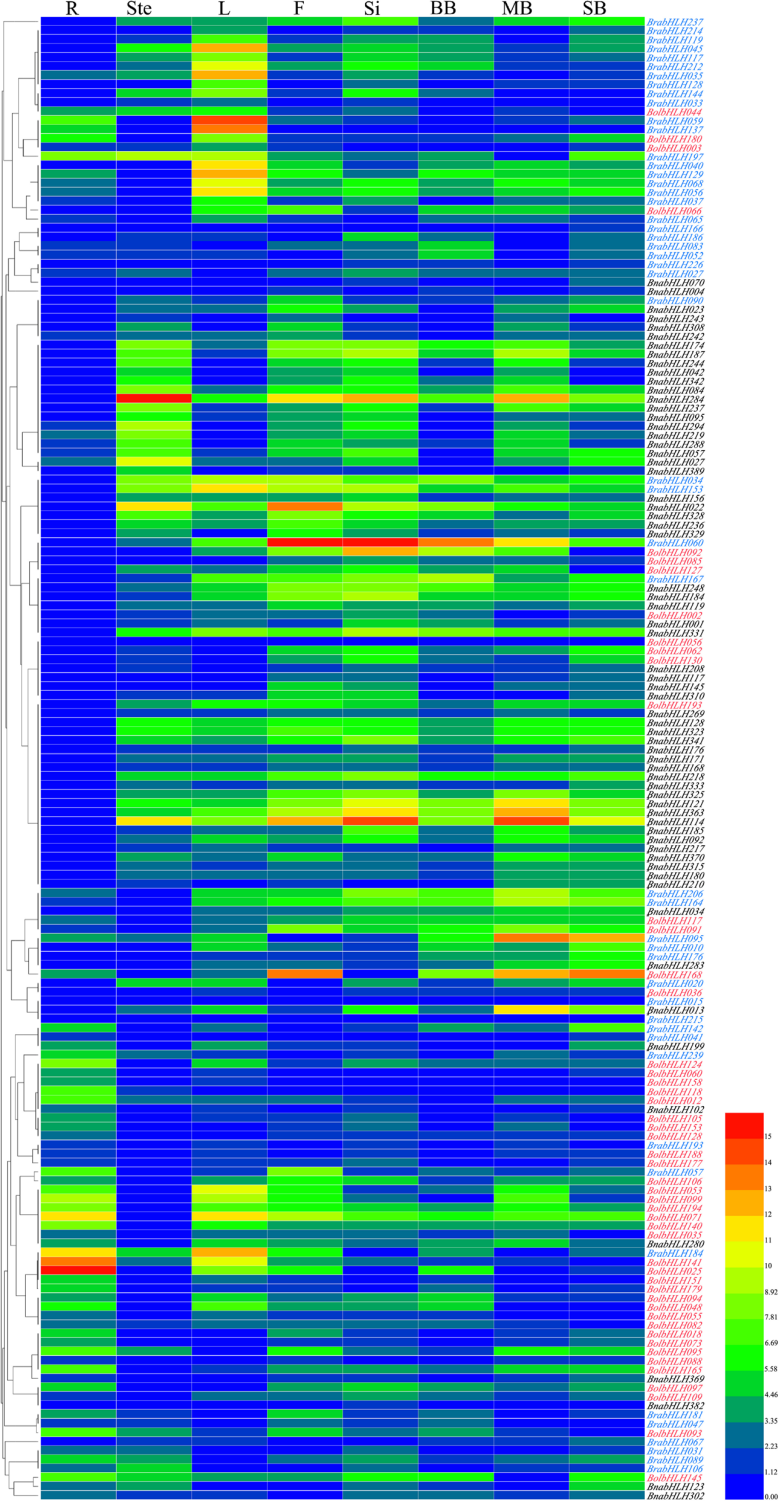
Expression patterns of 50 *BolbHLH*, 50 *BrabHLH*, and 65 *BnabHLH* genes in different tissues. All values underwent logarithmic transformation. R: root, Ste.: stem, L: leaves, F: flower, Si: silique, LB: large bud, MB: middle buds, and SB: small bud

To study whether the expression patterns of genes changed after the formation of tetraploid AACC genomes from the AA and CC genomes, the orthologous genes were selected for further research. The heat map showed that the expression patterns of bHLH genes in different species were diverse. However, some similarity or complementarity was observed. In *B. rapa* and *B. napus,* bHLH genes almost had no expression in the root. Nevertheless, their expression patterns in the stems, leaves, and reproductive organs were complementary. Most of genes had high expression in the stem, flower, silique, and floral buds and low expression in *B. napus* leaves, while *BrabHLH* genes had high expression in the leaves and low expression in the other tissues. Most of genes were highly expressed in *B. oleracea* roots, which was the opposite that in *B. rapa* and *B. napus*. Analyzing the temporal and spatial expression patterns of orthologous genes showed no significant similarity or complementarity between them (Fig. 9). For the newly identified *BrabHLH* genes, we analyzed the expression patterns of the two newly identified *BrabHLH* genes (*BrabHLH238* and *BrabHLH242* belonged to subfamily X) and found that they had the opposite expression pattern. *BrabHLH238* had no expression in the root and high expression in the stem, leaf, flower, silique, and flower buds. Meanwhile, *BrabHLH242* had a slightly high expression in the root and low expression in other tissues. We also analyzed which genes were specifically expressed in certain tissues or organs, and found that 9 genes in *B. rapa* were specifically expressed in leaves, 1 gene in *B. oleracea* was specifically expressed in roots, and 1 gene in *B. napus* was specifically expressed in floral buds.

### Gene expression analysis of some bHLH genes under hormone treatments

**Fig. 10:**
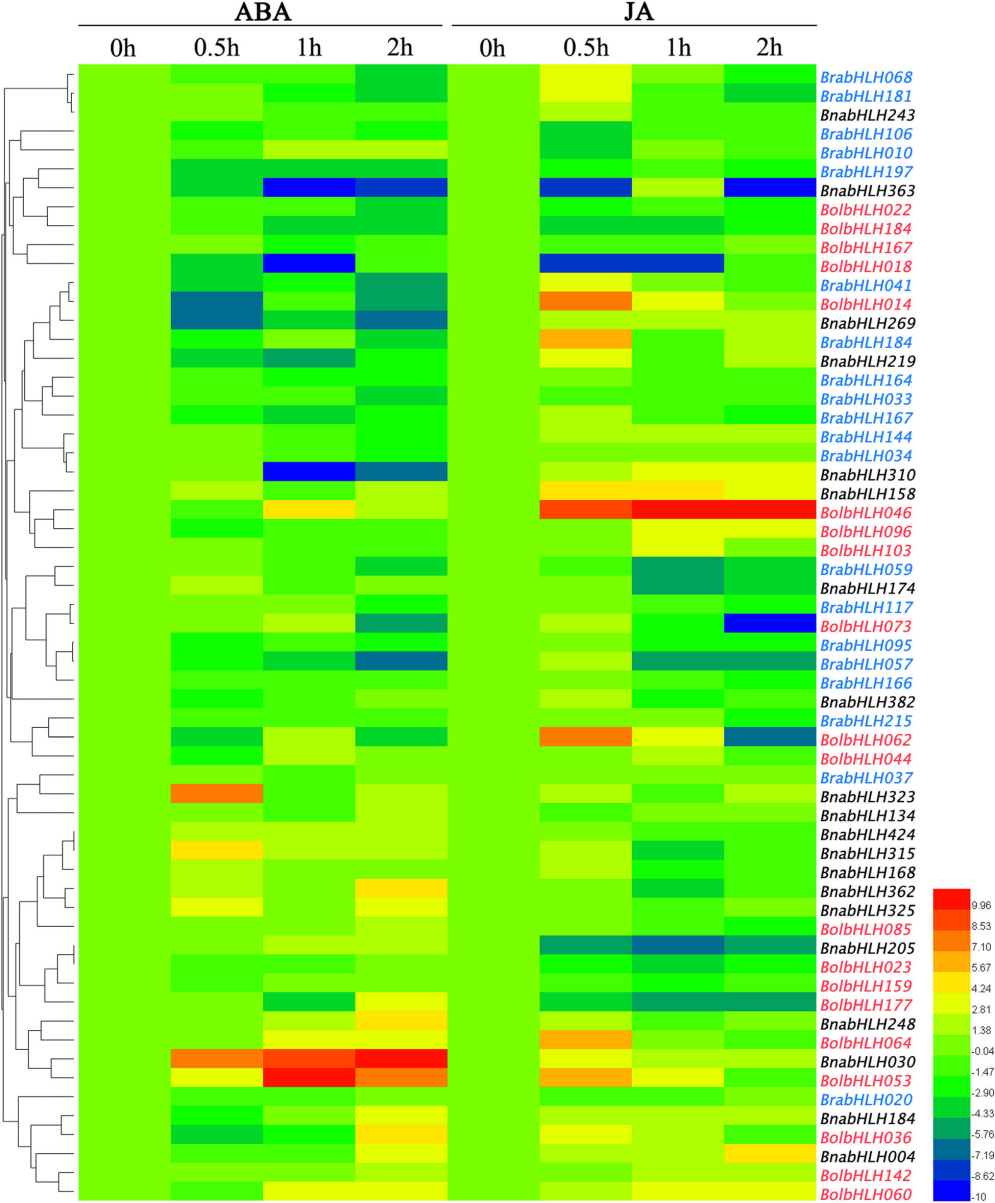
Expression analysis of 20 *BolbHLH*, 20 *BrabHLH* and 20 *BnabHLH* genes under ABA and JA treatments

Totally, 20 *BrabHLH*, 20 *BolbHLH* and 20 *BnabHLH* genes have randomly been chosen to conduct gene expression analysis after ABA and JA treatments. The leaves sampled at 0 h after treatments were used as the control and the relative expression of others samples were calculated (Table S10). All the relative expression results underwent logarithmic transformation and visualized with a heat map (Fig. 10). For ABA treatment, the expression levels of 24 genes decreased after treatment, including 13 *BrabHLH* genes, 4 *BnabHLH* genes and 7 *BolbHLH* genes. The expression levels of 16 genes were increased in, not in *B. rapa*, 10 in *B. napus* and 6 in *B. olerace*. The expression levels increased first and then decreased of 4 *BrabHLH* genes, 2 *BnabHLH* genes and 1 *BolbHLH* gene. There were 13 genes of decreased expression followed by increased expression, including 2 *BrabHLH* genes, 3 *BnabHLH* genes and 8 *BolbHLH* genes. Among these genes, *BnabHLH030*, *BnabHLH363*, *BnabHLH310*, *BnabHLH323*, *BolbHLH018* and *BolbHLH053* have a more dramatic change in expression levels. For JA treatment, there were 17 genes of decreased expression, including 7 *BrabHLH* genes, 2 *BnabHLH* genes and 8 *BolbHLH* gene. The expression levels of 1 *BrabHLH* gene, 7 *BnabHLH* genes and 6 *BolbHLH* genes increased after treatment, *BolbHLH0468* and *BolbHLH014* have large reduction; *BrabHLH037*, *BrabHLH020*, *BnabHLH134* and *BrabHLH034* have no obvious change. In addition, the expression levels of another genes were iterative processes, among them, the expression of *BolbHLH062* increased rapidly at 0.5 h after treatment, and then decreased. Totally, the number of down regulation genes was significantly more than the number of up regulation genes.

## Discussion

### bHLH gene family has been identified in many species

*Brassica* plant is an important economic crop in the world, and *B. oleracea* is a widely known species. With the rapid development of bioinformatics analysis, many genome sequences of *Brassica* plants have been completed at present [1, 25, 26]. Several gene families have been identified in *Brassica* to date [27–29]. After the discovery of bHLH motif with DNA binding and dimerization, increasing bHLH protein super families have been identified in plants and animals [4]. In animals, bHLH proteins are divided into six main group (groups A to F) according to their phylogenetic relationship, motifs, and functions [6]. A total of 230 bHLH genes have been identified in *B. rapa*, and the expression patterns of some genes with different treatments have also been analyzed [23]. In this study, 21 new bHLH genes in *B. rapa* were identified, due to the different versions of reference genomes.

### The bHLH genes have been selected during the evolution process

*A. thaliana* has experienced three whole genome duplication (WGD) events, as follows: a γ event shared with most dicots and two subsequent genome duplications (α and β) shared with other members of the order Brassicales [30]. *B. oleracea* has a common ancestor with *A. thaliana* and also underwent WGD events. At approximately 1.3–1.7 MYA, *B. oleracea* experienced a whole genome triplication (WGT) event, thereby resulting in the divergence of the genome between *B. oleracea* and *A. thaliana* [31, 32]. In this study, we found that divergence time of the bHLH gene family between the three *Brassica* crops and *A. thaliana* was approximately in the range of 1.0–1.3 MYA, which is close to the result of a previous study (1.3–1.7 MYA) [33]. To reveal the selection mechanism of bHLH genes in *B. oleracea*, *B. rapa* and *B. napu* during evolution, we calculated the *Ka*/*Ks* value, and most homologous gene pairs experienced purifying selection, thus indicating that these genes were strongly controlled in evolution. Only 12, 7, and 9 orthologous gene pairs had a *Ka*/*Ks* ratio of > 1 (Table S5, S6, S7), thereby suggesting that novel functions were likely to generate among these genes. Similar findings were observed in other gene families in plants, such as the TCS of tomato and PMEI of *Brassica*, of which most homolog pairs evolve through purifying selection, and a few or even no gene experiences positive selection [34, 35].

Theoretically, after WGD and WGT events, the genome size of *B. oleracea* should be threefold larger than that of *A. thaliana*. However, the size was much smaller than the theoretical value. The retention rate of *AtbHLH* orthologous genes was 57% in *B. oleracea*, which was close to its retention rate (56%) in *B. rapa*, while the actual retention rates in *B. rapa* and *B. oleracea* were 51.6 and 55.1%. The retention rate of *BolbHLH* was close to that of the core and random genes and slightly higher than that of the other gene families, such as the PMEI (52%) [35] and SDG gene families (43%) in *B. rapa* [36]. These results of gene retentions indicated that almost half of the genes were lost after WGT event. In *B. napus*, the retention rates of *AtbHLH* orthologous, core and random genes were low. This finding suggested that about half of genes have been lost during the formation of allotetraploid procedure.

### The expression patterns of bHLH genes provide some clues to study their functional analysis

bHLH gene family is an extremely large family that is involved in many regulation processes, such as stress response [37] and seed development [17, 38]. The heat map showed that the expression patterns of selected 50 *BolbHLH* genes were diverse. Although the functional studies on the function of bHLH genes in *B. oleracea* were still lacking, their gene function could be deduced *via* their expression patterns and also the reported gene functions of *A. thaliana* bHLH genes in the same subfamilies. *INDEHISCENT*, *HECATE*, and *SPATULA* are involved in the pistil development, and they belong to the VIIIb and I (a + b) subfamily, respectively [14, 15]. Our analysis of the expression patterns of *BolbHLH* genes belonging to the same subfamilies showed that these genes had relatively high expression in the reproductive organs, indicating their potential functions in reproductive development. After WGT, a series of events, such as chromosome rearrangement, gene loss, and epigenetic modification, often emerges [39, 40]. The remaining genes after the loss events are often associated with dosage effects or with new or subfunctionalized genes in *A. thaliana* [41, 42]. Homologous genes with similar expression patterns are preserved because of the dosage effect, while homologous genes with different expression patterns are retained due to new functionalization and subfunctionalization [43]. In this study, *BolbHLH098*/*BolbHLH155*, *BolbHLH154*/*BolbHLH226*, *BolbHLH141*/*BolbHLH090*, *BolbHLH133*/ *BolbHLH159*, and *BolbHLH198*/*BolbHLH167* were homologous genes, and 4 gene pairs had similar expression pattern, except for *BolbHLH141*/*BolbHLH090*. According to the heat map, most of *BolbHLH* genes had similar expression patterns (Fig. 9). According to the results above, we speculated that the retention of bHLH family genes was mainly due to the dosage effect in *B. oleracea*. In our study, bHLH genes had high expression in *B. oleracea* leaves, while they had high expression in reproduction tissues in *B. napus*. The main economic organ of *B. oleracea* was the leaf, while that of *B. napus* was the seed. The different expression patterns may be correlated with the agronomic traits during the evolution course. In some studies, researchers have found that bHLH genes are related to plant biomass. For example, *OsbHLH107* overexpression can enhance the grain size in rice, and a *Vitis vinifera* bHLH transcription factor (*VvCEB1*(opt)) enhances plant cell size, vegetative biomass, and reproductive yield [44, 45]. We compared the expression patterns of the specifically expressed genes between the *A. thaliana* and the three *Brassica* crops combined with their gene structures. The results indicated that these orthologous gene pairs have similar gene structures, while their expression patterns were diverse (Data not given). This means that their expression patterns are not related to their conserved gene structures.

The expression levels of some bHLH genes were analyzed after ABA and JA treatments. Some genes respond to ABA or JA among the selected genes in this study and the expressions of *BolbHLH064* and *BnabHLH030* changed simultaneously after ABA and JA treatments. *OsbHLH006* (*RERJ1*), a jasmonic acid-responsive bHLH gene, response to drought stress and belong to III(a + c) subfamily [3, 10, 23]. *BrabHLH041*, *BolbHLH142* and *BnabHLH174* of the the genes we chosen belong to III(a + c) subfamily, the expression level of *BrabHLH041*, *BolbHLH142* and *BnabHLH174* changed after ABA and JA treatments. This indicated that the expression analysis will give some help to study their biological functions.

### GO annotation of *BolbHLH* genes indicated their functions

An important regulation pathway is that transcription factors interact with *cis*-acting elements to express the genes involved in stress response and developmental processes specifically. In the present study, the functional annotation of the *BolbHLH* genes showed that they were mainly concentrated in the DNA-binding transcription factor, DNA binding, transcription regulator, and protein dimerization activities in the molecular function section, which was consistent with the way in which the bHLH gene functions by forming homodimers or heterodimers [4]. bHLH transcription factors can regulate many genes involved in different regulatory pathways [46]. bHLH transcription factors also participate in the regulation of metabolic processes, such as the biosynthesis of alkaloids and nicotine [47]. bHLH genes are also involved in plant response to biotic and abiotic stresses. *TcMYC* is highly expressed in the xylem and leaves and upregulated by drought and high-salinity stresses in yew trees [48]. The *CsbHLH18* of sweet orange functions in the modulation of cold tolerance by regulating the antioxidant gene [49]. Some bHLH transcription factors can also respond to salinity and Fe-deficient abiotic stress [50, 51]. In the present study, the biological process of GO annotation showed that most of genes had the GO terms of cellular N compound, metabolic process, biosynthetic process, anatomical structure development, response to stress, regulation of transcription, DNA template, and reproduction. These enriched GO annotations were also consistent with the known functional bHLH transcription factors in some studies.

## Conclusion

In this study, we identified and performed the comparative genomics analysis of bHLH gene family among *B. oleracea*, *B. rapa* and *B. napus* and also investigated their diversity. The expression patterns between *B. rapa* and *B. napus* shows that they have the similar expression pattern in the root and opposite patterns in the stems, leaves, and reproduction tissues. Further analysis demonstrated that some bHLH gene members may play a crucial role under the abiotic and biotic stress conditions. This is the first to report on the bHLH gene family in *B. oleracea* and *B. napus.* These findings can offer useful information on the functional analysis of the bHLH genes in plants.

## Methods

### Identification of bHLH family genes in *B. oleracea*, *B. rapa* and *B. napus*

To identify bHLH family genes in *B. oleracea* and *B. napus*, we searched the protein sequences of the 162 reported *Arabidopsis* bHLH from TAIR (http://www.arabidopsis.org/) and downloaded the protein sequences of 230 bHLH genes that were identified in *B. rapa* from the *Brassica* Database (http://brassicadb.org/brad/index.php) according to previous studies [5, 23, 52, 53]. We obtained the candidate genes of bHLH in *B. oleracea* and *B. napus* by analyzing the amino acid sequences of *A. thaliana* and *B. rapa* bHLH family members with BLASTP in the *Brassica* Database (E value = 10^− 5^). We also searched all the orthologous genes of 162 *A. thaliana* bHLH genes. Data of syntenic relationship between *A. thaliana* and different *Brassica* crops (like *B. rapa*, *B. oleracea*, *B. napus* and some others) are provided in *Brassica* database. After entering the *Brassica* database, select “Syntenic gene” under the menu of “Search”, and then operate according to the step1 and step2 indicated on the page to obtain the orthologous genes of *A. thaliana* in different *Brassica* crops. The reference genome of *B. oleracea* and *B. napus* were provided to *Brassica* Database by Dr. Liu and Dr. Chalhoub, respectively [1, 54]. Then, we downloaded the amino acid sequences of all candidate genes, and the Pfam database (E value = 1.0, http://pfam.xfam.org/) was used to determine whether each sequence harbored the conserved domains (HLH, PF00010.25). Genes that did not contain the known conserved domains of the gene families were excluded from further analysis. All amino acid sequences were subjected to gene ontology (GO) annotation by using Blast2GO with default parameters [55].

### Chromosomal localization of bHLH family genes in *B. oleracea*, *B. rapa* and *B. napus*

We searched the start and stop locations on the *B. oleracea*, *B. rapa* and *B. napus* chromosomes of all bHLH family members in the *Brassica* database. Considering the numerous members in *B. napus*, the analysis of *B. napus* was divided into AA and CC genomes and the subsequent analyses were in the same way. Subsequently, the chromosomal localization of these members was performed using the MapChart software on the basis of the relative location of each gene on each chromosome [56].

### Gene structure and phylogenetic analyses of bHLH family genes in *B. oleracea, B. rapa* and *B. napus*

We searched the full-length DNA and cDNA sequences of *B. oleracea*, *B. rapa* and *B. napus* bHLH genes from the National Center for Biotechnology Information, (https://www.ncbi.nlm.nih.gov/). Then, the Gene Structure Display Server database (http://gsds.cbi.pku.edu.cn/index.php) was utilized to analyze the gene structure. The homologous sequence alignment of all bHLH genes from *B. oleracea*, *B. rapa* and *B. napus* was performed using ClustalW [57]. Then, the sequence alignment results were considered the basis in generating the unrooted phylogenetic tree of the *B. oleracea*, *B. rapa* and *B. napus* bHLH genes with MEGA (version 5.0) [58]. All parameters used were default parameters. Phylogenetic trees were generated with the value of the 1000 bootstrap samples by the neighbor-joining (NJ) method [58]. Then, the results of gene structure analysis were integrated with phylogenetic trees by using Photoshop CS3.

### Nonsynonymous substitution rate, synonymous substitution rate, and gene retention analysis

The nonsynonymous substitution rates (*Ka*) and synonymous substitution rates (*Ks*) of the orthologous bHLH gene pairs among the three *Brassica* crops and *A. thaliana* were calculated. First, the multiple sequence alignments of the CDS sequence pairs were performed using Clustalx [59]. Then, the values of *Ka* and *Ks* were figures out by *Ka/Ks*_Calculator software with the files of sequence alignment, the method YN was chosen to do calculation [60]. The relationships of orthologous genes between *A. thaliana* and *B. oleracea* were also visualized using the Circos program [61].

The retention rates of *Arabidopsis* bHLH, core, and random genes and the retention rate in the different subgenomes (i.e., LF, MF1, and MF2) of *B. rapa*, *B. oleracea*, and *B. napus* (AA and CC genomes) were counted. The gene loci with tandem repeats were calculated using one gene. The 458 core genes and 459 random genes of *A. thaliana* were downloaded from the CEGMA database (http://korflab.ucdavis.edu/Datasets/cegma); the homologous genes of 917 genes in *B. rapa*, *B. oleracea* and *B. napus* were searched in BRAD, and their retention rates were counted [62, 63]. The retention and loss of genes on the AA and CC genome in *B. napus* during tetraploid were analyzed.

### Motif analysis, signal peptide, and subcellular localization prediction in *B. oleracea, B. rapa* and *B. napus*

MEME was used to analyze the common conserved the short amino acid sequence of bHLH family members [64]. In this study, we searched 15 bHLH gene motifs. Then, the conserved motifs were integrated with phylogenetic trees by using Photoshop CS3. SignalP-4.1 (http://www.cbs.dtu.dk/services/SignalP/) was utilized to predict the signal peptide of the bHLH genes in *B. oleracea, B. rapa* and *B. napus*, all of which are set at default [65]. We can predict the position of signal peptide according to the values of C, S and Y in the results. Each amino acid corresponds to one S value, and the value of signal peptide region is higher; Each amino acid has a C value, and the highest C value is the shear site. Y-max value was used to predict the shear site, where S value is the steep position and the site with high C value.

In order to confirm the predicted results more reliable, three prediction methods were used to predict the subcellular localization of bHLH gene. They are WolfPsort (https://wolfpsort.hgc.jp) [66], Plant-mPLoc (http://www.csbio.sjtu.edu.cn/bioinf/plant-multi/) [67] and ProtComp 9.0 (http://linux1.softberry.com). If two or more of the predicted results are identical, it is regarded as the subcellular localization of the encoded protein. BrabHLH051, BrabHLH245, BolbHLH207 and BnabHLH024 were randomly selected for homeopathic expression in tobacco cells. The CDS sequences of these genes were amplified from *B. rapa*, *B. oleracea* and *B. napus* with a high-fidelity enzyme (Vazyme, China). The primers were seen in Table S11. We constructed the CDS sequences on the pFGC vector containing GPF fluorescence marker by homologous recombination. After that, the constructed vectors were injected into tobacco leaves, and the GFP fluorescence signal in tobacco leaf cells was observed by laser confocal microscope LSM780 (ZEISS, Germany) after 36 h of growth.

### Plant material, hormone treatments and expression pattern analyses of bHLH genes in *B. oleracea, B. rapa* and *B. napus*

The plant materials (*B. rapa* accession Chiifu-401-42 and *B. napus* cv. Zheyou 606) for expression patterns analyses were planted in the experimental farm of Zhejiang University. *B. oleracea* cv. Zhegan No. 1 was grown in the experimental farm of Zhejiang Academy of Agricultural Science. All plant materials were not deposited in a publicly available herbarium. Roots, stems, leaves, flowers, silique, big bud (> 2.0 mm), middle buds, and small bud (< 2.0 mm) in the flowering period were sampled in liquid nitrogen and stored in a refrigerator at − 80 °C. The plant materials (*B. rapa*, *B. oleracea* and *B. napus*) for hormone treatments were planted in the glass greenhouse. The leaves of the three *Brassica* crops were treated with ABA and JA. When the plants grew five true leaves, the leaves were sprayed with 100 μM ABA and 100 μM JA, and samples were collected at 0 h, 0.5 h, 1 h and 2 h after treatments. All samples were frozen in liquid nitrogen and stored in a refrigerator at − 80 °C.

Total RNA was extracted from previous materials by using the TRIzol reagent (Invitrogen, USA) following the manufacturer’s instructions. The first cDNA strand was generated following the manufacturer’s protocol by using the Takara Reverse Transcription System (Japan). A total of 50 *B. oleracea*, 50 *B. rapa*, and 65 *B. napus* bHLH genes were chosen to analyze the expression patterns, and these genes were orthologous genes. The primers used for qRT-PCR in *B. oleracea*, *B. rapa* and *B. napus* are shown in Table S12, S13, and S14, respectively. We selected 20 genes from each crop and analyzed their expression patterns after treatments. The primers were designed using Integrated DNA Technologies (https://sg.idtdna.com/pages) and the Primer (version 5.0) software. All cDNA samples were adjusted to a uniform concentration. SYBR Green Master Mix Reagent (TOYOBO, Japan) were used to do the Real-time fluorescent quantitative PCR. Three technical replicates for each sample were performed in a real-time PCR machine (Bio-Rad CFX Manager). To normalize the total amount of cDNA present in each reaction, we amplified the gene *UBC10* of *B. rapa*, *GAPDH* of *B. oleracea* and *25S* of *B. napu* as endogenous controls in calibrating the relative expression [68–70]. The 2^−ΔΔCT^ method of the relative gene quantification recommended by Applied Biosystems (PE Applied Biosystems, USA) was used to calculate the expression level of different tissues [71]. For expression patterns analyses, we set the tissue with the lowest gene expression as the control, calibrated the expression value to 1, and calculated the relative gene expression in other tissues. For the treatments, the samples collected at 0 h after treatments were set as control. The relative expression were taken as a logarithmic form of all genes and make a heat map with Helm software [72].

## Supplementary information

**Additional file 1: Figure S1.** Phylogenetic tree of bHLH genes of *B. oleracea*, *B. rapa*, *B. napus* and *A. thaliana.* Branches of the bHLH genes in *A. thaliana* and *B. rapa* were labeled in red and green, respectively. The numbers on the branches indicate the bootstrap percentage values calculated from 1000 replicates.

**Additional file 2: Figure S2.** Phylogenetic tree of *B. oleracea* bHLH genes with domain sequences. The numbers on the branches indicate the bootstrap percentage values calculated from 1000 replicates.

**Additional file 3: Figure S3.** Phylogenetic tree of bHLH genes of AA genome of *B. napus*. The numbers on the branches indicate the bootstrap percentage values calculated from 1000 replicates.

**Additional file 4: Figure S4.** Phylogenetic tree of bHLH genes of CC genome of *B. napus*. The numbers on the branches indicate the bootstrap percentage values calculated from 1000 replicates.

**Additional file 5: Figure S5.** Phylogenetic tree of *B. rapa* bHLH genes with domain sequences. The numbers on the branches indicate the bootstrap percentage values calculated from 1000 replicates.

**Additional file 6: Figure S6.** Sequence logos of bHLH protein motifs of the three *Brassica* crops. Logos are a visualization tool for motifs. The height of a letter indicates its relative frequency at the given position.

**Additional file 7: Figure S7.** Conserved motifs analyses of bHLH genes in *B. oleracea*

**Additional file 8: Figure S8.** Conserved motifs analyses of bHLH genes in *B. rapa*

**Additional file 9: Figure S9.** Conserved motifs analyses of bHLH genes in *B. napus*. A: The conserved motifs of AA genome of *B. napus*; B: The conserved motifs of CC genome of *B. napus.*

**Additional file 10: Figure S10.** Gene structure analyses of bHLH genes in *B. oleracea*. Exons and introns are represented by boxes and lines, respectively.

**Additional file 11: Figure S11.** Gene structure analyses of bHLH genes in *B. rapa*. Exons and introns are represented by boxes and lines, respectively.

**Additional file 12: Figure S12.** Gene structure analyses of bHLH genes in the *B. napus*. A: Exons- introns ananalyses of bHLH genes in AA genome of *B. napus*; B: Exons- introns ananalyses of bHLH genes in CC genome of *B. napus*. Exons and introns are represented by boxes and lines, respectively.

**Additional file 13: Figure S13.** Signal peptide prediction of BolbHLH128 (A), BrabHLH084 (B), and BrabHLH168 (C)

**Additional file 14: Table S1.** Identified bHLH genes in *B. oleracea* and their molecular characteristics

**Additional file 15: Table S2.** Identified bHLH genes in the AA genome of *B. napus* and their molecular characteristic

**Additional file 16: Table S3.** Identified bHLH genes in the CC genome of *B. napus* and their molecular characteristics

**Additional file 17: Table S4.** Identified bHLH genes in *B. rapa* with 21 newly identified *BrabHLH* genes and their molecular characteristics. The genes with yellow background were newly identified *BrabHLH* genes in this study, while the others were identified in a previous study (Song et al., 2014).

**Additional file 18: Table S5.** Nonsynonymous and synonymous substitution rates of orthologous bHLH genes between *B. oleracea* and *A. thaliana*

**Additional file 19: Table S6.** Nonsynonymous and synonymous substitution rates of orthologous bHLH genes between *A. thaliana* and *B. rapa*

**Additional file 20: Table S7.** Nonsynonymous and synonymous substitution rates of orthologous bHLH genes in *B. napus*

**Additional file 21: Table S8.** Subcellular location predictions of partial bHLH proteins

**Additional file 22: Table S9.** Original Ct values of selected genes of *B. olerecea*, *B. rapa* and *B. napus*

**Additional file 23 Table S10.** Relative expression levels of 20 *BolbHLH*, 20 *BrabHLH*, and 20 *BnabHLH* genes under ABA and JA treatments

**Additional file 24: Table S11.** Primer sequences of selected genes for subcellular localization analysis

**Additional file 25 Table S12.** Primers of selected bHLH genes for qRT-PCR in *B. oleracea*

**Additional file 26: Table S13.** Primers of selected bHLH genes for qRT-PCR in *B. rapa*

**Additional file 27: Table S14.** Primers of selected bHLH genes for qRT-PCR in *B. napus*

## Data Availability

All sequences used in this study can be found in *Brassica* Database (http://brassicadb.org/brad/) according to their gene ID numbers. In addition, the genome sequences of *B. oleracea* used for identifying the bHLH genes in this study were deposited in the DDBJ/EMBL/GenBank nucleotide core database under the accession code AOIX00000000; The genome sequences of *B. rapa* used for identifying the bHLH genes were deposited at DDBJ/EMBL/GenBank under the accession AENI00000000; Sequence Read Archive accession numbers of *B. napus* sequencing data are ERP005275 and PRJEB6069, which been used to identify the bHLH genes in *B. napus*. The other data sets generated in this study are included within the article and supplementary files. All materials used or generated during the study are kept in our laboratory and are available from the corresponding author by reasonable request.

## Abbreviations

bHLH: Basic helix–loop–helix
CDS: Coding sequence
*HEC*: *HECATE*
*IND*: *INDEHISCENT*
MYA: Millions of years ago
ORF: Open reading frame
PMEI: Pectin methylesterase inhibitors
SDG: SET domain group
*SPT*: *SPATULA*
TCS: Two component signaling system
WGD: Whole genome duplication
WGT: Whole genome triplication
