# Supplementary figures and images for: Comparative analysis of basic helix–loop–helix gene family among Brassica oleracea, Brassica rapa, and Brassica napus
Source: BMC Genomics. 2020 Feb 24;21:178. doi: 10.1186/s12864-020-6572-6 (PMC7041300; doi:10.1186/s12864-020-6572-6)

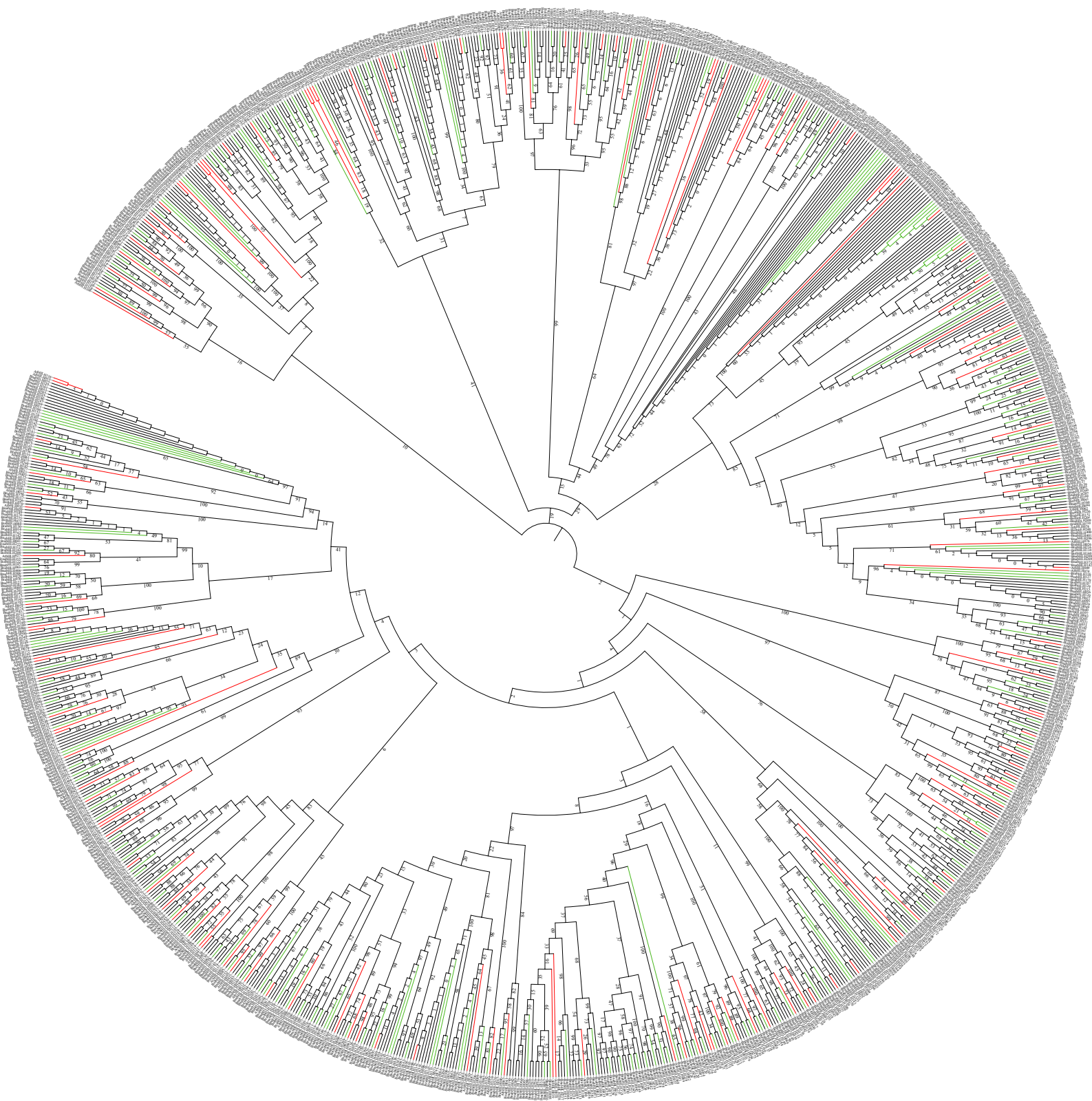

Supplement: Supplementary file 1 — Additional file 1: Figure S1. Phylogenetic tree of bHLH genes of B. oleracea, B. rapa, B. napus and A. thaliana. Branches of the bHLH genes in A. thaliana and B. rapa were labeled in red and green, respectively. The numbers on the branches indicate the bootstrap percentage values calculated from 1000 replicates. [file 12864_2020_6572_MOESM1_ESM.pdf]

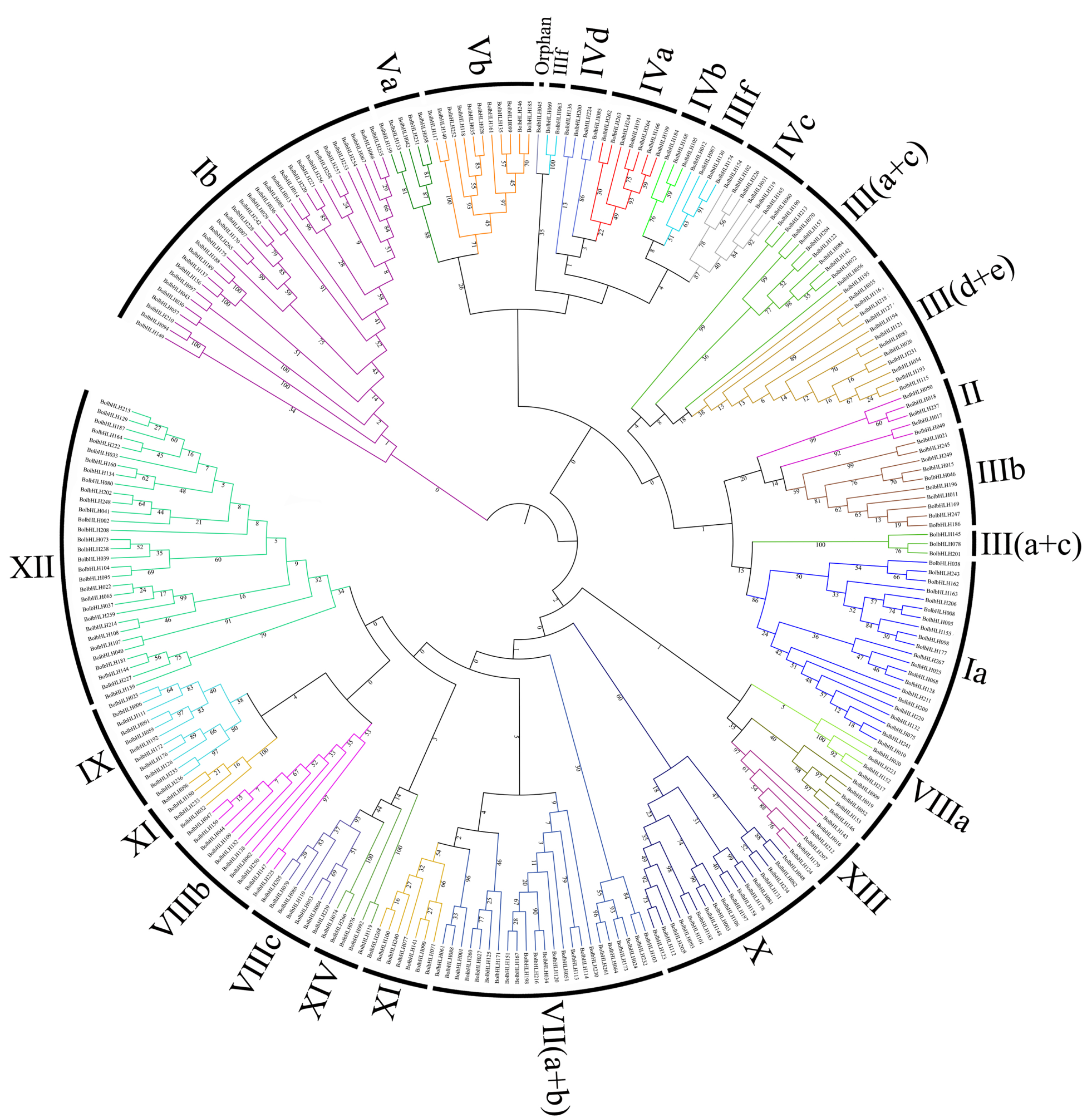

Supplement: Supplementary file 2 — Additional file 2: Figure S2. Phylogenetic tree of B. oleracea bHLH genes with domain sequences. The numbers on the branches indicate the bootstrap percentage values calculated from 1000 replicates. [file 12864_2020_6572_MOESM2_ESM.pdf]

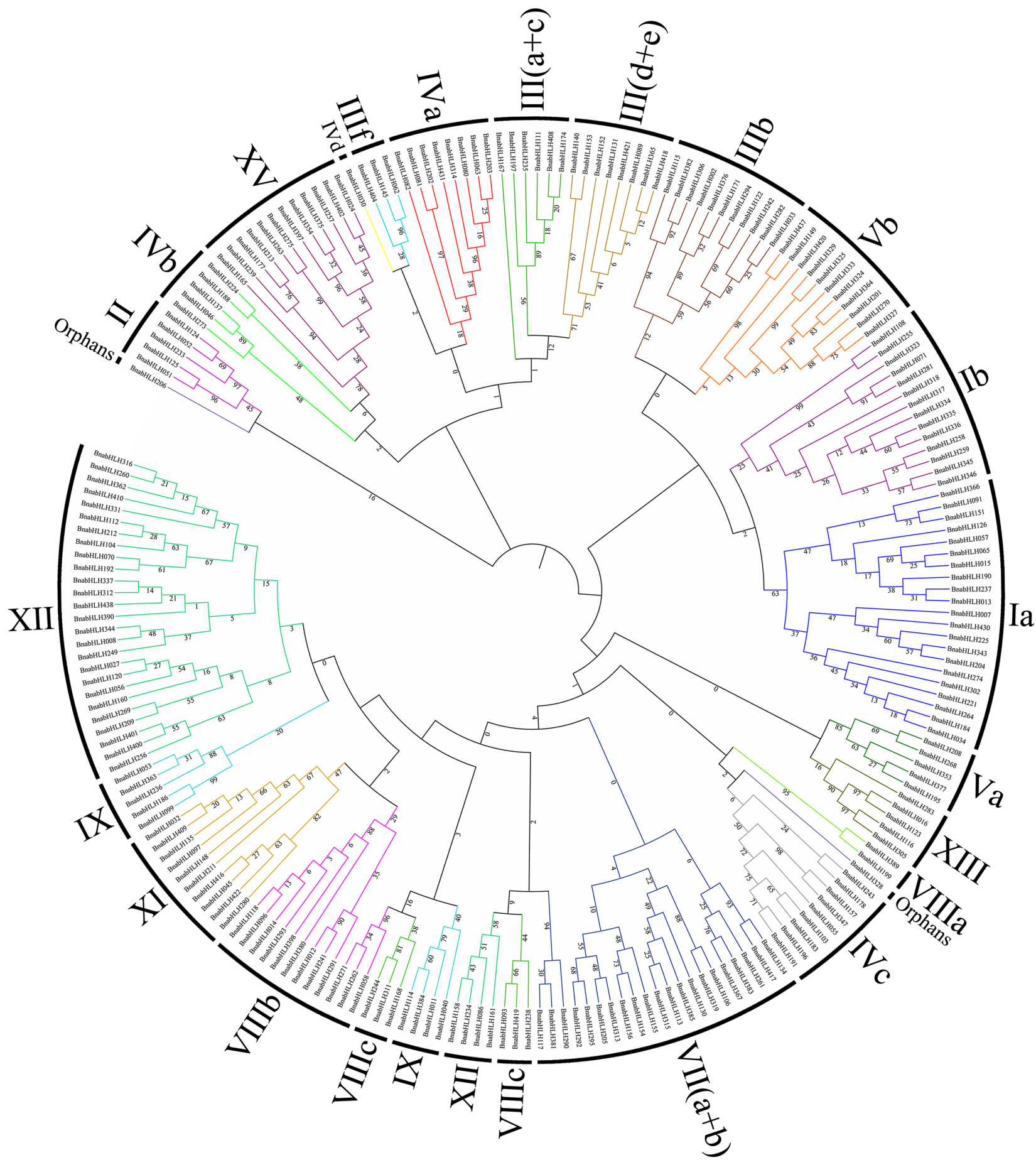

Supplement: Supplementary file 3 — Additional file 3: Figure S3. Phylogenetic tree of bHLH genes of AA genome of B. napus. The numbers on the branches indicate the bootstrap percentage values calculated from 1000 replicates. [file 12864_2020_6572_MOESM3_ESM.pdf]

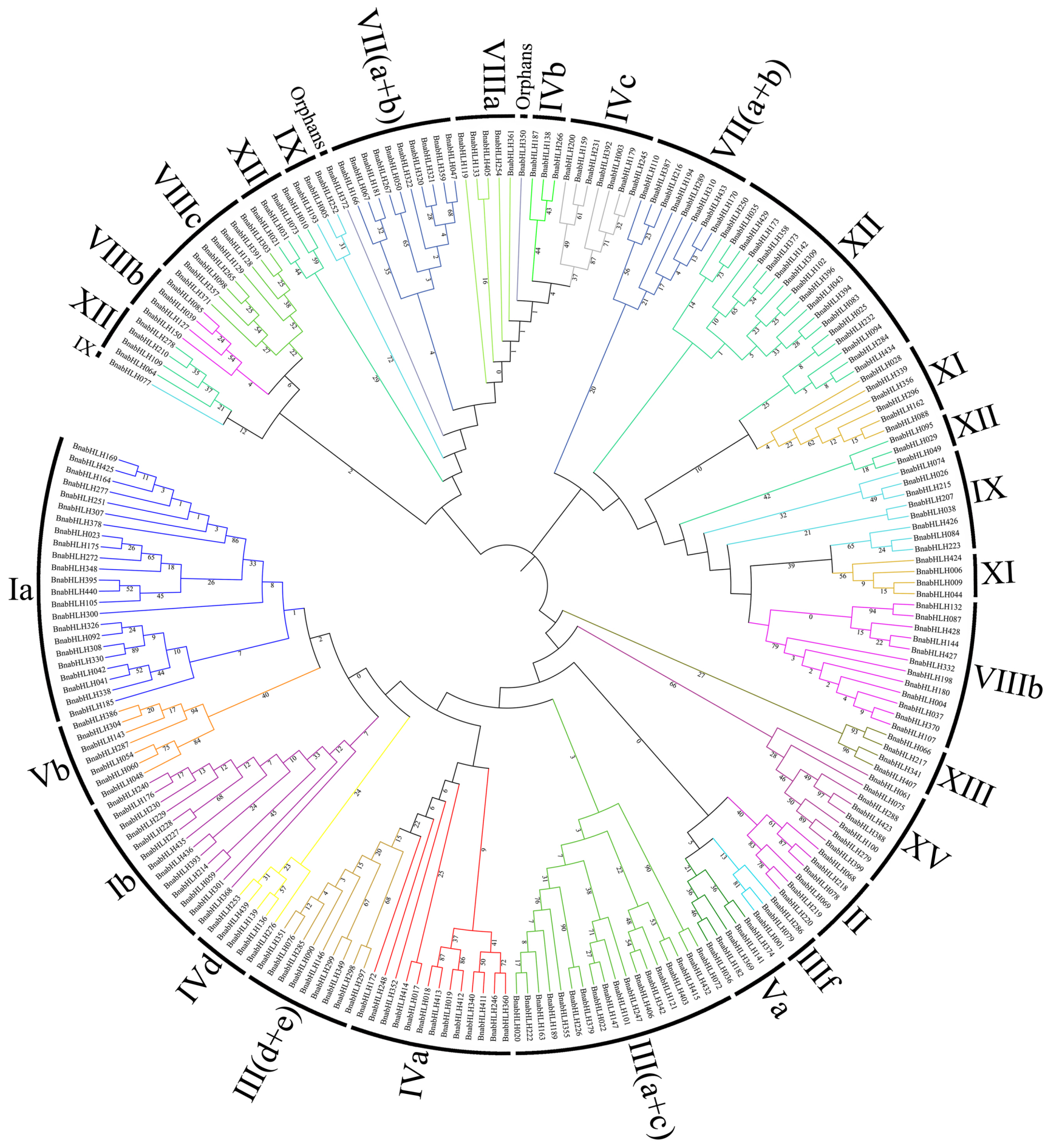

Supplement: Supplementary file 4 — Additional file 4: Figure S4. Phylogenetic tree of bHLH genes of CC genome of B. napus. The numbers on the branches indicate the bootstrap percentage values calculated from 1000 replicates. [file 12864_2020_6572_MOESM4_ESM.pdf]

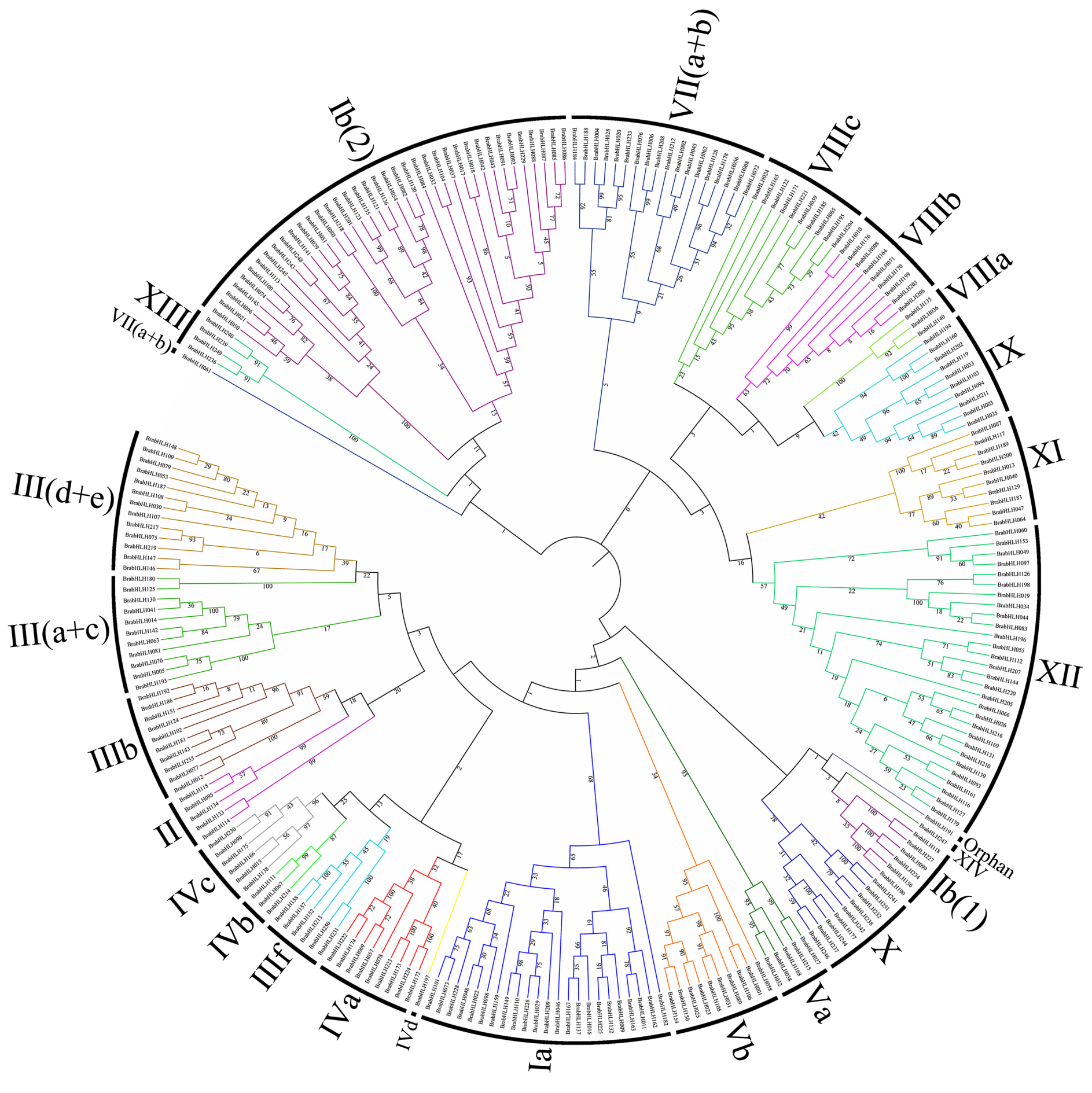

Supplement: Supplementary file 5 — Additional file 5: Figure S5. Phylogenetic tree of B. rapa bHLH genes with domain sequences. The numbers on the branches indicate the bootstrap percentage values calculated from 1000 replicates. [file 12864_2020_6572_MOESM5_ESM.pdf]

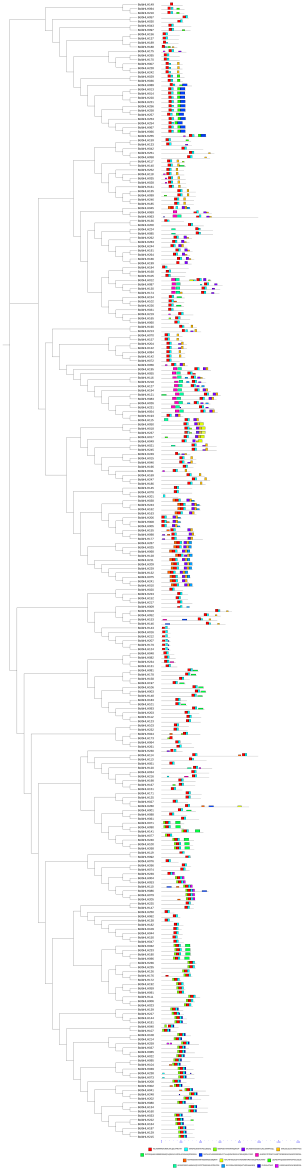

Supplement: Supplementary file 7 — Additional file 7: Figure S7. Conserved motifs analyses of bHLH genes in B. oleracea [file 12864_2020_6572_MOESM7_ESM.pdf]

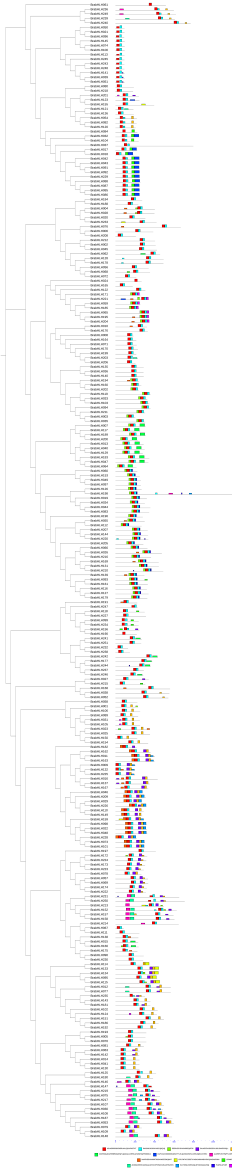

Supplement: Supplementary file 8 — Additional file 8: Figure S8. Conserved motifs analyses of bHLH genes in B. rapa [file 12864_2020_6572_MOESM8_ESM.pdf]

A

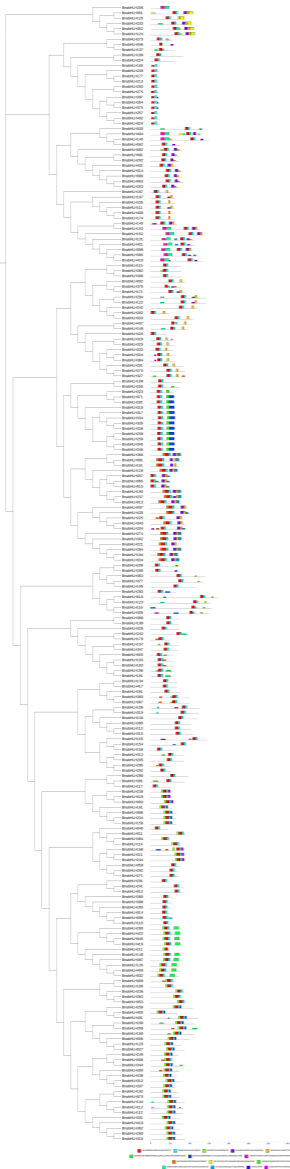

B

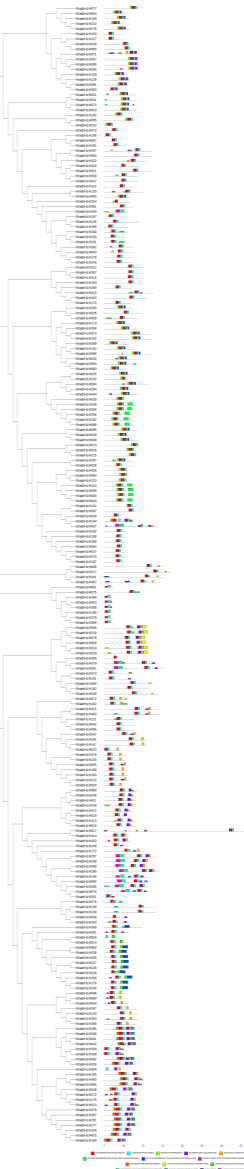

Supplement: Supplementary file 9 — Additional file 9: Figure S9. Conserved motifs analyses of bHLH genes in B. napus. A: The conserved motifs of AA genome of B. napus; B: The conserved motifs of CC genome of B. napus. [file 12864_2020_6572_MOESM9_ESM.pdf]

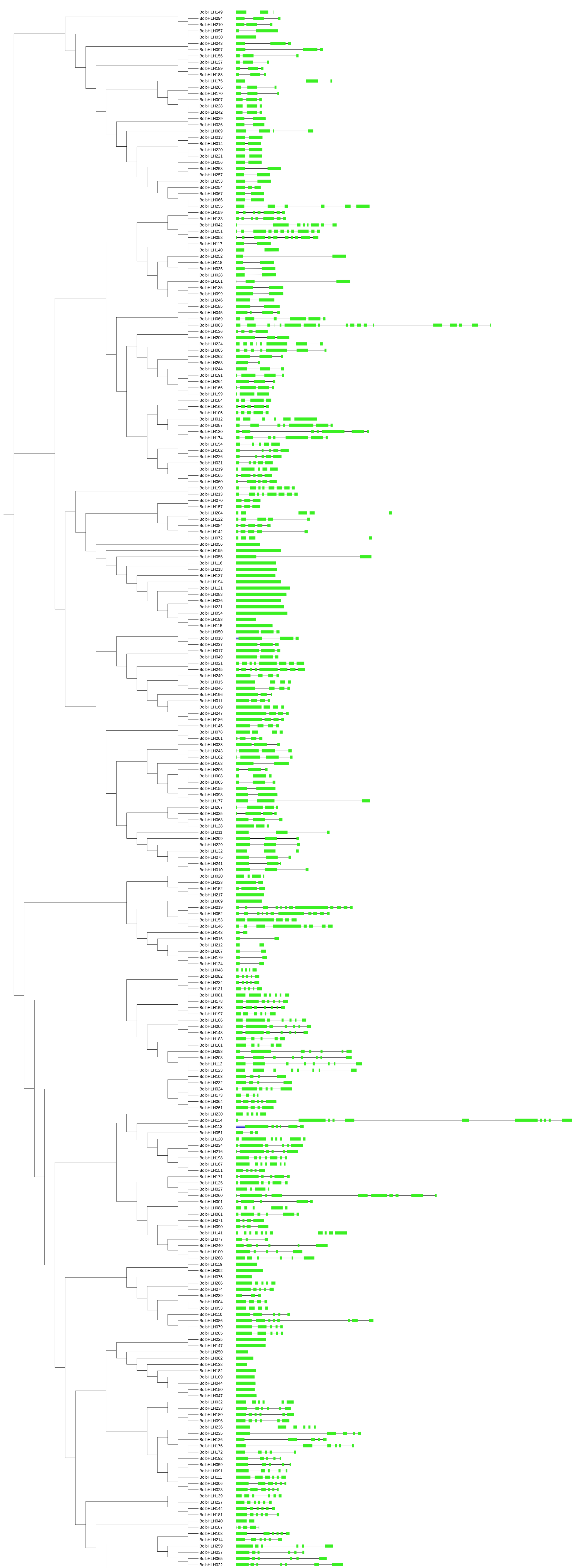

Supplement: Supplementary file 10 — Additional file 10: Figure S10. Gene structure analyses of bHLH genes in B. oleracea. Exons and introns are represented by boxes and lines, respectively. [file 12864_2020_6572_MOESM10_ESM.pdf]

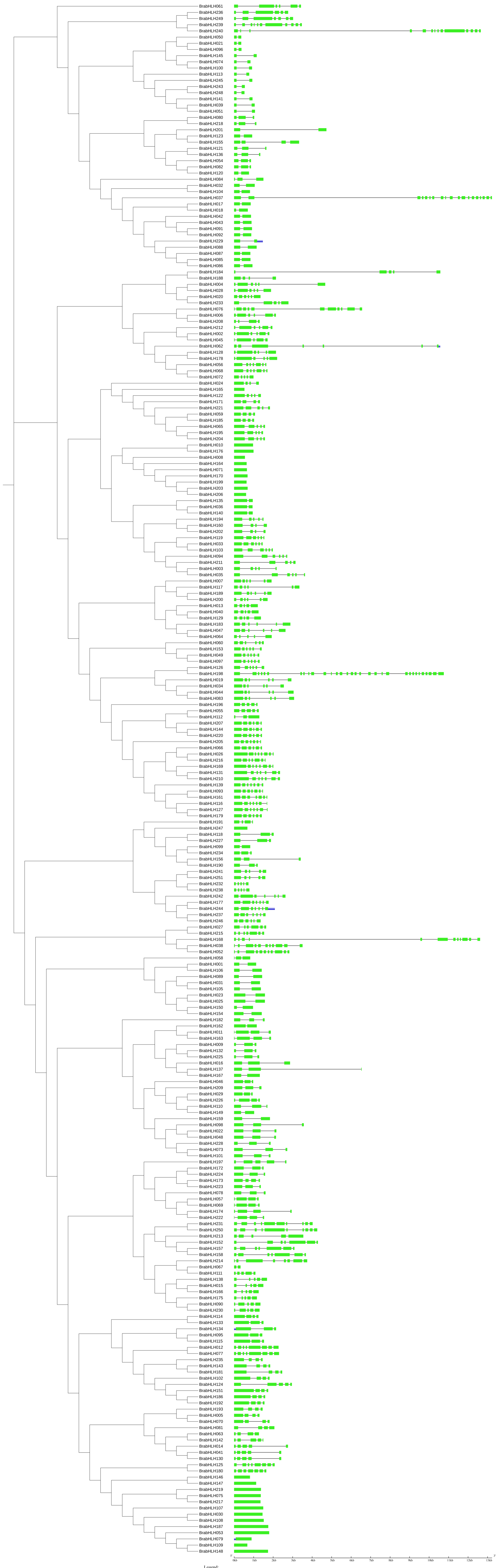

Supplement: Supplementary file 11 — Additional file 11: Figure S11. Gene structure analyses of bHLH genes in B. rapa. Exons and introns are represented by boxes and lines, respectively. [file 12864_2020_6572_MOESM11_ESM.pdf]

A

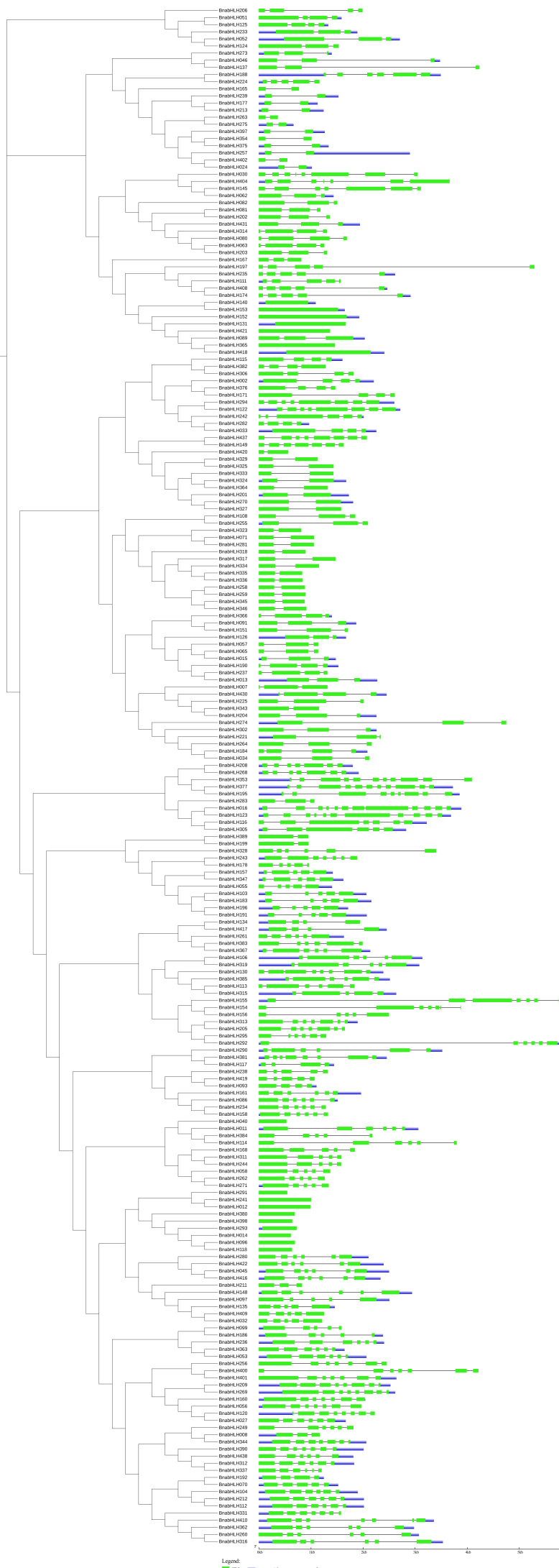

B

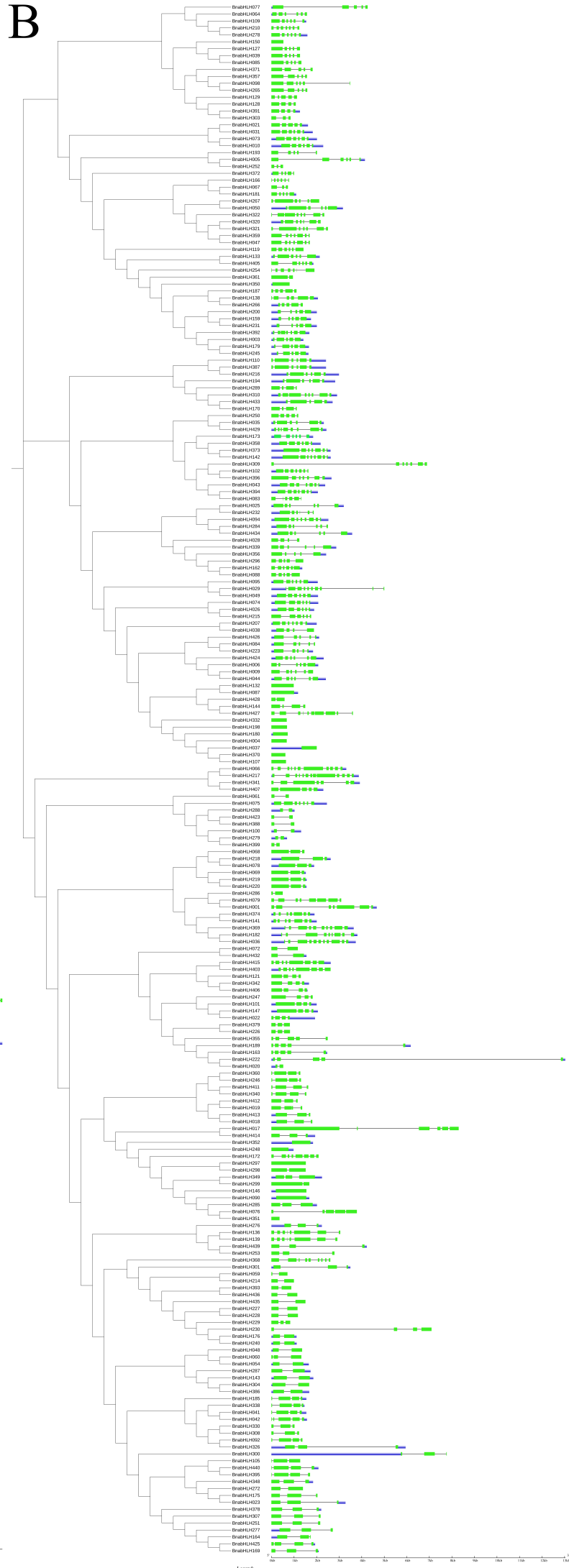

Supplement: Supplementary file 12 — Additional file 12: Figure S12. Gene structure analyses of bHLH genes in the B. napus. A: Exons- introns ananalyses of bHLH genes in AA genome of B. napus; B: Exons- introns ananalyses of bHLH genes in CC genome of B. napus. Exons and introns are represented by boxes and lines, respectively. [file 12864_2020_6572_MOESM12_ESM.pdf]

A # SignalP-4.1 euk predictions  
>BolbHLH128

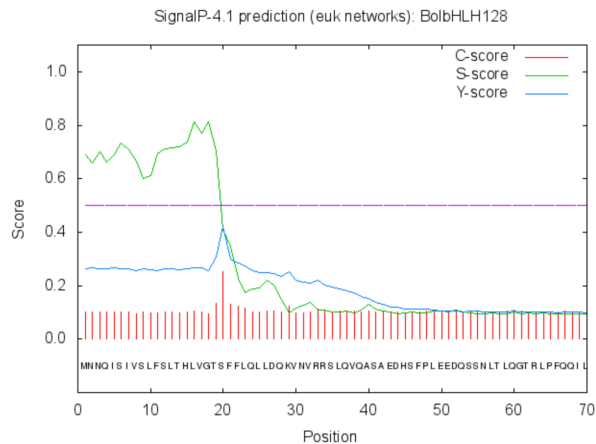

B >BrabHLH084

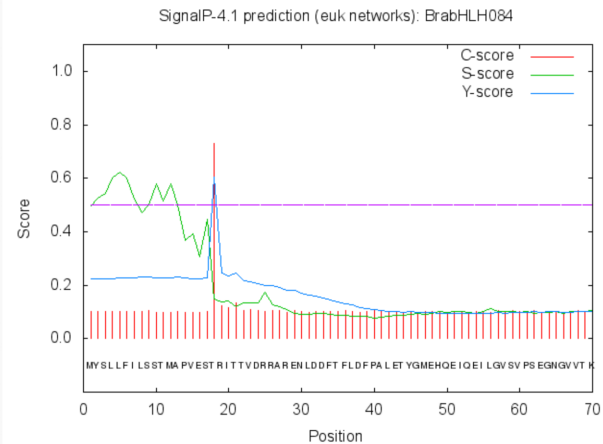

C >BrabHLH168

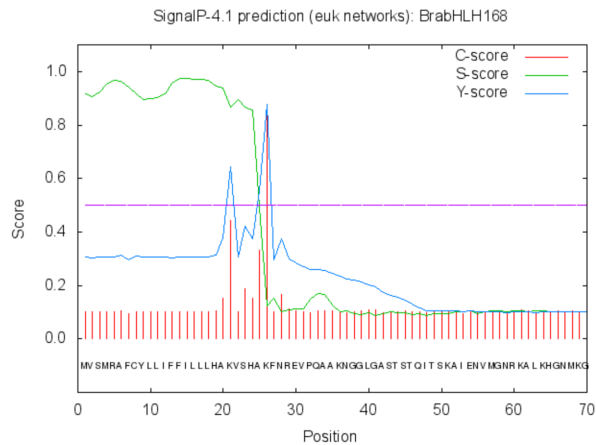

Supplement: Supplementary file 13 — Additional file 13: Figure S13. Signal peptide prediction of BolbHLH128 (A), BrabHLH084 (B), and BrabHLH168 (C) [file 12864_2020_6572_MOESM13_ESM.pdf]
