# Supplementary material for: Comparative analysis of basic helix–loop–helix gene family among Brassica oleracea, Brassica rapa, and Brassica napus
Source: BMC Genomics. 2020 Feb 24;21:178. doi: 10.1186/s12864-020-6572-6 (PMC7041300; doi:10.1186/s12864-020-6572-6)
Supplement: Supplementary file 6 — Additional file 6: Figure S6. Sequence logos of bHLH protein motifs of the three Brassica crops. Logos are a visualization tool for motifs. The height of a letter indicates its relative frequency at the given position. [file 12864_2020_6572_MOESM6_ESM.pdf]

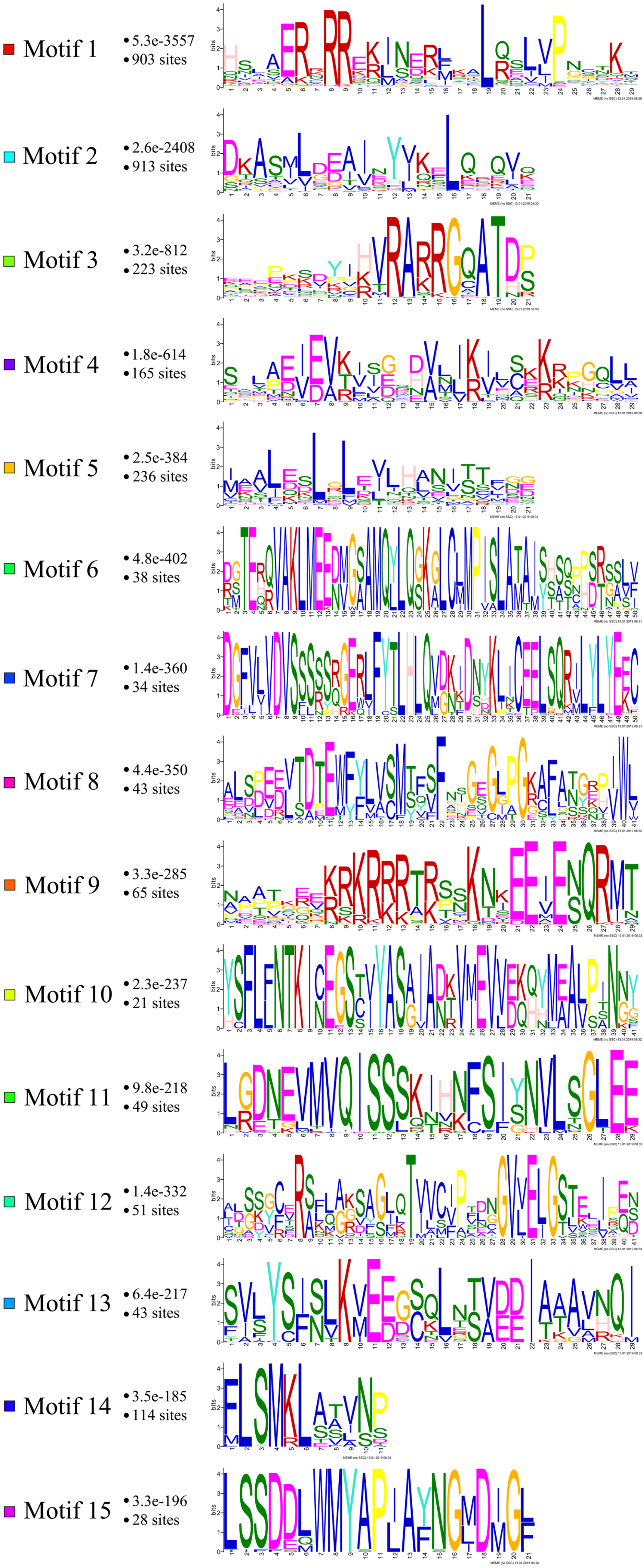
Motif 1
5.3e-3557
903 sites
Motif 2
2.6e-2408
913 sites
Motif 3
3.2e-812
223 sites
Motif 4
1.8e-614
165 sites
Motif 5
2.5e-384
236 sites
Motif 6
4.8e-402
38 sites
Motif 7
1.4e-360
34 sites
Motif 8
4.4e-350
43 sites
Motif 9
3.3e-285
65 sites
Motif 10
2.3e-237
21 sites
Motif 11
9.8e-218
49 sites
Motif 12
1.4e-332
51 sites
Motif 13
6.4e-217
43 sites
Motif 14
3.5e-185
114 sites
Motif 15
3.3e-196
28 sites
